# Glycolytic suppression dramatically changes the intracellular metabolic profile of multiple cancer cell lines in a mitochondrial metabolism-dependent manner

**DOI:** 10.1038/s41598-019-55296-3

**Published:** 2019-12-10

**Authors:** Reika Shiratori, Kenta Furuichi, Masashi Yamaguchi, Natsumi Miyazaki, Haruna Aoki, Hiroji Chibana, Kousei Ito, Shigeki Aoki

**Affiliations:** 1 Laboratory of Biopharmaceutics, Graduate School of Pharmaceutical Sciences, Chiba University, Inohana 1-8-1, Chuo-ku, Chiba-city, Chiba 260-8675 Japan; 2 Medical Mycology Research Center, Chiba University, Inohana 1-8-1, Chuo-ku, Chiba-city, Chiba 260-8673 Japan

**Keywords:** Cancer metabolism, Cancer metabolism, Cancer therapy, Cancer therapy

## Abstract

Most cancer cells rely on glycolysis to generate ATP, even when oxygen is available. However, merely inhibiting the glycolysis is insufficient for the eradication of cancer cells. One main reason for this is that cancer cells have the potential to adapt their metabolism to their environmental conditions. In this study, we investigated how cancer cells modify their intracellular metabolism when glycolysis is suppressed, using PANC-1 pancreatic cancer cells and two other solid tumor cell lines, A549 and HeLa. Our study revealed that glycolytically suppressed cells upregulated mitochondrial function and relied on oxidative phosphorylation (OXPHOS) to obtain the ATP necessary for their survival. Dynamic changes in intracellular metabolic profiles were also observed, reflected by the reduced levels of TCA cycle intermediates and elevated levels of most amino acids. Glutamine and glutamate were important for this metabolic reprogramming, as these were largely consumed by influx into the TCA cycle when the glycolytic pathway was suppressed. During the reprogramming process, activated autophagy was involved in modulating mitochondrial function. We conclude that upon glycolytic suppression in multiple types of tumor cells, intracellular energy metabolism is reprogrammed toward mitochondrial OXPHOS in an autophagy-dependent manner to ensure cellular survival.

## Introduction

Normal mammalian cells obtain ATP, an energy source that is necessary for survival, from both glycolysis and mitochondrial oxidative phosphorylation (OXPHOS). On the other hand, cancer cells mainly obtain ATP from glycolysis rather than OXPHOS, even in the presence of sufficient oxygen^1^. However, mitochondrial respiration is upregulated when the tricarboxylic acid (TCA) cycle is activated in non-small cell lung cancer, glioblastoma, and breast cancer cells, suggesting that mitochondrial function is not necessarily defective in cancer cells^2^. Nevertheless, most cancer cells rely on glycolysis, which accounts for about 60% of total ATP production^3^. Glycolysis, which ultimately produces lactate, generates two ATP molecules per glucose molecule, and is thus less efficient than OXPHOS, which generates about 36 ATPs per glucose^1^. On the other hand, glycolysis can generate ATP faster than OXPHOS^4^. Therefore, increased glycolysis is beneficial for rapid growth and proliferation of cancer cells. However, one of the main reasons why cancer cells upregulate the glycolytic pathway is to provide glycolytic intermediates, which are necessary for biosynthesis of nucleic acids, proteins, and lipids, as well as for generation of lactate to maintain the NAD^+^/NADH redox balance^5^. Extensive studies have gradually clarified the mechanisms by which cancer cells upregulate glycolysis at the molecular level. One key component of this process is hypoxia-inducible factor 1α (HIF-1α), which induces transcription of glycolytic enzymes such as hexokinase and lactate dehydrogenase A (LDHA)^6^. In addition, HIF-1 inactivates pyruvate dehydrogenase (PDH), which converts pyruvate into acetyl CoA, via activation of PDH kinase-1 (PDK-1), resulting in suppression of the TCA cycle^7^. Therefore, upregulating glycolysis rather than OXPHOS confers many survival advantages on cancer cells.

Cancer therapies have been developed that target transporters and enzymes in the glycolytic pathway, such as glucose transporter 1 (GLUT1), hexokinase, and LDHA^8^. For instance, inhibition of GLUT1 by WZB117 prevents the growth of human lung cancer cells both *in vitro* and *in vivo*^9,10^. The hexokinase inhibitors 2-deoxyglucose (2-DG) and 3-bromopyruvvate also suppress the proliferation of a variety of cancer cells *in vitro*^11,12^. However, monotherapy with hexokinase inhibitor has limited efficacy *in vivo*^13,14^, whereas 2-DG increases the ability of adriamycin and paclitaxel to decrease the volume of human tumors^13,14^. Based on these reports, we can speculate that merely inhibiting glycolysis only suppresses growth, but is insufficient for the eradication of cancer; by contrast, combination therapy using a glycolytic inhibitor with other anti-cancer drugs is more effective at suppressing tumors. Cancer cells can adapt their metabolism to survive when glycolysis is suppressed, and it is necessary to understand how cancer cells modulate their intracellular metabolism under these adverse conditions.

The metabolic pathways upregulated in cancer cells have been extensively studied. For instance, glutaminolysis, which supports biosynthesis and maintains bioenergetics and redox balance, is critical for cancer cell growth^15^. However, it remains unclear how cancer cells change their metabolism, as well as how exposure to anti-cancer drugs affects the overall metabolism. To address such issues, we can take advantage of metabolomics, a rapidly developing suit of approaches that permit comprehensive analysis. In particular, metabolomics focusing on water-soluble metabolites, including tumor-promoting metabolic pathways such as the glycolysis and the TCA cycle, has already contributed to our understanding of cancer metabolism^16^. Capillary electrophoresis-time-of-flight mass spectrometry (CE-TOFMS) has been recognized as a useful tool for the global analysis of water-soluble charged metabolites^17,18^. CE-MS has extremely high resolution and can be used to analyze almost any charged species^19^. CE-TOFMS, in which a high-speed scanning and high-resolution time-of-flight mass spectrometer is connected to a CE apparatus, can perform high-throughput quantitative analysis with precise mass resolution^20^. Therefore, we can use CE-TOFMS to obtain global information about when and how the levels of each metabolite change. Soga’s group used CE-TOFMS–based metabolomics to clearly show that glycolytic metabolites are significantly less abundant in colon and stomach cancer tissues than in normal tissues^17^. Interestingly, in those cancer tissues, the levels of most amino acids were elevated, suggesting that the metabolomic approach can reveal specific metabolic profiles of cancer cells. Furthermore, Soga’s group clarified that the protooncoprotein MYC regulates multiple metabolic pathways, including pyrimidine metabolism, in colorectal cancer^21^, suggesting that new therapeutic strategies targeting these metabolic pathways could be effective. Therefore, metabolomics can provide valuable insight into metabolic pathways in cancer, as well as facilitate the development of therapeutic strategies targeting those pathways.

In recent years, combination therapies in which anti-cancer agents are used with other drugs have been tested in clinical trials and applied in clinical practice. For instance, in a randomized phase II trial, the anti-diabetic agent metformin improved progression-free survival relative to several chemotherapies (cisplatin, epirubicin hydrochloride, capecitabine, and gemcitabine hydrochloride) in patients with metastatic pancreatic cancer^22^. In another clinical trial, the Bcl-2 inhibitor venetoclax exhibited synergistic anti-cancer effects with ibrutinib, and this combination therapy has been approved for treatment of chronic lymphocytic leukemia^23^. Although it was initially largely unclear why such combination therapies are effective, recent works have partially elucidated the mechanisms underlying these synergistic effects by focusing on alterations in cellular metabolism. As a representative example, in melanoma, myelogenous leukemia, renal cancer, and colorectal cancer cells, the combination of tyrosine kinase inhibitors with biguanide agents prevents cell growth by suppressing synthesis of amino acids and biguanide-induced reductive glutamine metabolism^24^. In addition, in a previous study, we showed that metformin increases sensitivity to imatinib, a tyrosine kinase inhibitor, by altering intracellular metabolic signaling in chronic myelogenous leukemia cells^25^. Another group demonstrated that the combination of venetoclax and the hypomethylating agent azacytidine targets leukemia stem cells in patients with acute myeloid leukemia; the drugs exert their effect by decreasing mitochondrial OXPHOS through disruption of the TCA cycle^26^. To develop more effective combination therapies altering cancer metabolism, it would be useful to improve our understanding of cancer cell metabolism.

Recently, the mechanisms of compensatory energy acquisition pathways in glycolysis-suppressed cancer cells have been intensively explored. For instance, several types of cancer cells can reprogram metabolism toward other pathways, including mitochondrial OXPHOS, in order to survive when glycolysis is suppressed^27–29^. However, the regulatory mechanisms underlying these shifts in cancer metabolism are not fully understood. In this study, we investigated how cancer cells acquire energy when glycolytic pathways are suppressed, using PANC-1 pancreatic cancer cells and two other solid tumor cell lines, A549 and HeLa. We found that glycolytic suppression upregulated mitochondrial function and altered the metabolic profile, in particular the metabolites of the TCA cycle and amino acids. In addition, glycolytic suppression induced autophagy, thereby increasing the amino acid supply and promoting maintenance of mitochondrial function in glycolysis-suppressed cells. Our findings suggest that intracellular glycometabolism is reprogrammed to use mitochondrial OXPHOS via autophagy when glycolysis is suppressed. Our results provide insight into appropriate strategies for combination therapy targeting cancer metabolism in multiple types of cancer cells.

## Results

### Glycolytic suppression dynamically changes the glycometabolism profile of PANC-1 cells

**Figure 1:**
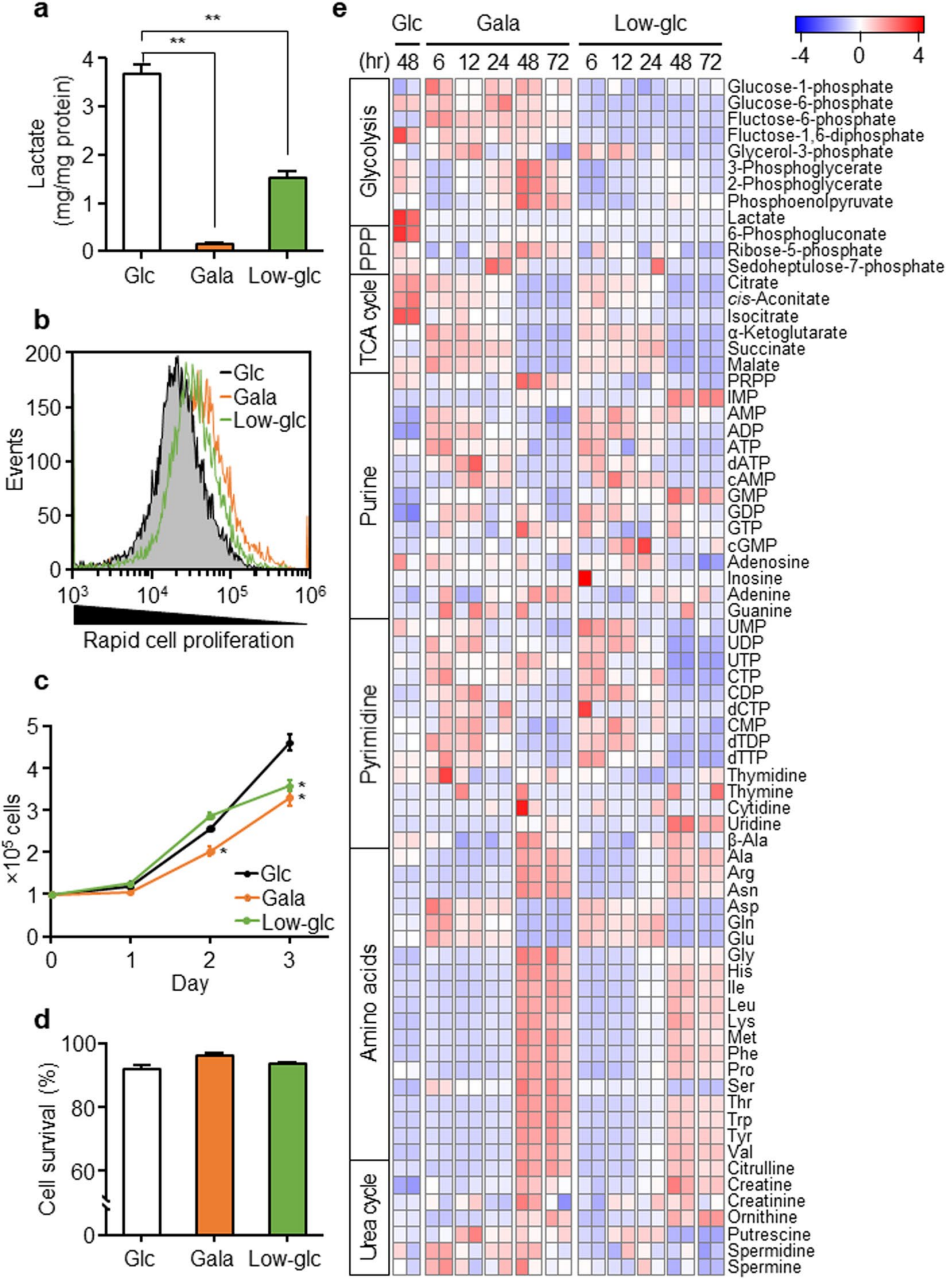
Glycolytic suppression dynamically changes the glycometabolism profile of PANC-1 cells. (**a**) PANC-1 cells were cultured in glucose (Glc), galactose (Gala), or low-glucose (Low-glc) medium for 72 hr. The amount of lactate released into the medium from the cells for the last 24 hr was measured. Data represent means ± SD of three independent cell cultures. **P < 0.01. (**b**) Proliferation of PANC-1 cells cultured in Glc, Gala, or Low-glc for 96 hr were evaluated by CFSE dye dilution using flow cytometry. Data are representative of three independent cell cultures. (**c**) PANC-1 cells were pre-cultured with Glc, Gala, or Low-glc for 48 hr, and then cell proliferation in each culture medium was determined by counting cells. Data represent means ± SD of three independent cell cultures. *P < 0.05, compared with cells cultured in Glc. **(d)** Survival rate of the cells in **(a)** was evaluated based on PI uptake, as determined by flow cytometry. Data represent means ± SD of three independent cell cultures. **(e)** The levels of metabolites extracted from PANC-1 cells cultured in Glc, Gala, or Low-glc medium were measured by CE-TOFMS and normalized against the amount of total cellular protein. Metabolomic patterns were visualized using z-score plots and heat maps. Data represent two independent cell cultures for each condition.

To examine the metabolic profiles of cancer cells when the glycolytic pathway was suppressed, we replaced the glucose in the culture medium with galactose or a low concentration of glucose (low-glucose); the conditions were based on our previous research^30^. Oxidation of galactose to pyruvate through glycolysis yields no net ATP^31^. Here, we investigated intracellular metabolism in PANC-1 human pancreatic cancer cells as a representative model of solid tumor cells. Replacement of sugar source dramatically decreased the release of lactate, the final product of the glycolysis, confirming that changing the sugar source in the culture medium suppressed glycolysis in PANC-1 cells (Fig. 1a). In this glycolysis-suppressed condition, we investigated cell proliferation by carboxyfluorescein diacetate succinimidyl ester (CFSE) staining. CFSE dye is retained within cells, and the fluorescence intensity is reduced by half in the daughter cells following each cell division. Fluorescence intensity was higher in cells cultured in galactose or low-glucose than in cells cultured in glucose (Fig. 1b). The reduced cell proliferation in PANC-1 cells cultured with galactose or low-glucose was quantitatively confirmed by growth curves based on cell numbers (Fig. 1c). However, merely suppressing glycolysis barely affected cell survival, as shown by the marginal increase in the percentage of propidium iodide (PI)-positive cells cultured in galactose or low-glucose (Fig. 1d). Taken together, these data indicate that glycolysis-suppressed PANC-1 cells could survive, although their growth was mildly suppressed.

Therefore, we speculated that another energy acquisition pathway helps PANC-1 cells to survive when the glycolytic pathway is inhibited. To investigate alternative pathways, we used CE-TOFMS to monitor intracellular metabolites related to glycometabolism in glycolysis-suppressed PANC-1 cells. CE-TOFMS-based metabolomics can analyze water-soluble charged metabolites, including the glycolytic products and intermediates of the TCA cycle, as well as amino acids. After the sugar source in the culture medium was changed from glucose to galactose or low-glucose, intracellular metabolic patterns gradually shifted over time; by 48 hr after replacement of the sugar source, the metabolic shift appeared to be complete (Fig. 1e). The level of lactate was dramatically decreased in cells cultured in galactose or low-glucose, confirming that glycolysis was suppressed. Interestingly, the levels of all intermediates of the TCA cycle were dramatically reduced in PANC-1 cells cultured in galactose or a low-glucose. On the other hand, the levels of amino acids (alanine, arginine, asparagine, glycine, histidine, isoleucine, leucine, lysine, methionine, phenylalanine, proline, serine, threonine, tryptophan, tyrosine, and valine) were increased by replacement of the sugar source, whereas the levels of glutamine, glutamate, and aspartate, all of which are also intermediates of the TCA cycle, were significantly reduced. In addition, quantitative proteomic analysis of PANC-1 cells cultured in medium containing galactose or low-glucose for 48 hr revealed that glycolytic suppression changed the levels of enzymes involved in the TCA cycle and OXPHOS (Supplementary Fig. S1a). In particular, the levels of isocitrate dehydrogenase (NAD(+)) 3 gamma (IDH3G), succinate-CoA ligase ADP-forming beta subunit (SUCLA2), and malate dehydrogenase 2 (MDH2), all of which are enzymes of the TCA cycle, were increased by glycolytic suppression. Also, the levels of OXPHOS enzymes, NADH-ubiquinone oxidoreductases, NDUFA (4, 5, 6 and 9), NDUFB (4, 7 and 10), NDUFS (2 and 7), NDUFV (1 and 2), ATPase (5H), and ATP synthases (6V1A, 6V1B2, and 6V1E1) were elevated in cells cultured with galactose. In low-glucose, the levels of ATPases (5B, 5E, 5O, 5D, and 5A1) and ATP synthases (6V0D1, 6V1C1, 6V1F, and 6V1D) were elevated. Amino acid-related enzymes such as asparagine synthetase (ASNS), ornithine aminotransferase (OAT), glutamic-oxaloacetic transaminase (GOT) 1, and glutamate dehydrogenase (GDH) 1 were also more abundant. The elevated levels of MDH2 and GDH1/2 in glycolysis-suppressed PANC-1 cells were confirmed by western blotting (Supplementary Fig. S1b). These results indicate that glycolysis-suppressed PANC-1 cells could maintain their survival and reprogram their intracellular metabolic pathways, and that mitochondria-dependent intracellular metabolism and amino acid metabolism changed dynamically.

### Glycolytic suppression results in upregulation of mitochondrial function, which maintains the survival of PANC-1 cells

**Figure 2:**
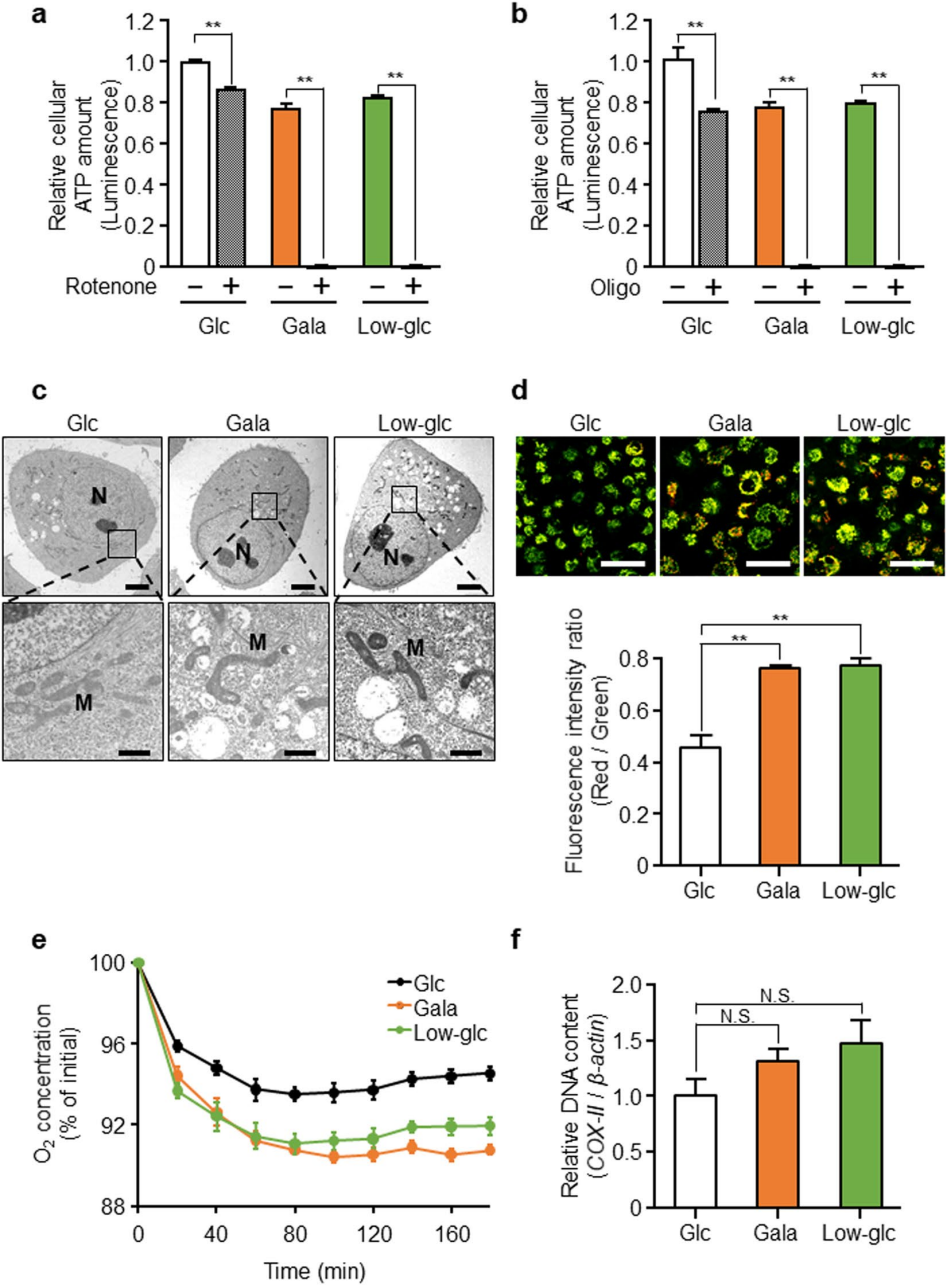
Glycolytic suppression results in upregulation of mitochondrial function, which maintains the survival of PANC-1 cells. **(a**,**b)** PANC-1 cells were cultured in glucose (Glc), galactose (Gala), or low-glucose (Low-glc) medium with or without rotenone (100 µM) **(a)** or oligomycin (Oligo; 20 ng/mL) **(b)** for 48 hr, and then intracellular ATP content was quantitated. The data were normalized against the level in PANC-1 cells cultured in Glc medium without rotenone or oligomycin. Data represent means ± SD of three independent cell cultures. **P < 0.01. (**c**) Intracellular structures in PANC-1 cells cultured in Glc, Gala, or Low-glc medium for 48 hr were observed using transmission electron microscopy. “N” indicates nucleus, and “M” indicates mitochondria. Scale bars, 5 µm (upper) or 1 µm (lower). (**d**) PANC-1 cells in (**c**) were stained with JC-1 and observed by confocal microscopy. Representative images show merged images of polymeric (red) and monomeric (green) JC-1. Scale bars, 50 µm. The ratio of polymeric to monomeric JC-1 (i.e., the ratio of fluorescence intensities) was calculated. Data represent means ± SD of three independent cell cultures. **P < 0.01. (**e**) The oxygen concentration in the culture medium of PANC-1 cells cultured in Glc, Gala, or Low-glc medium for 48 hr was measured over time. Data represent means ± SD of three independent cell cultures. (**f**) Copy number of mitochondrial DNA, reflected by the level of the mitochondrial gene *cytochrome c oxidase subunit II* (*COX-II*) in the cells in (**c**), was quantitated. Relative amounts of mitochondrial DNA in cells were calculated after normalizing against nuclear *β-actin* DNA. Data represent means ± SD of three independent cell cultures. N.S., not significant.

In general, mammalian cells generate ATP through two metabolic pathways, glycolysis and mitochondrial OXPHOS. To determine whether glycolytic suppression affects mitochondrial OXPHOS activity, we first evaluated the effect of OXPHOS inhibition on intracellular ATP production when glycolysis was suppressed in PANC-1 cells. To inhibit OXPHOS, we treated PANC-1 cells with rotenone and oligomycin, which inhibit complex I and complex V (ATP synthase) of the mitochondrial electron transport chain, respectively. We found that the glycolysis-suppressed cells were highly sensitive to these inhibitors, which dramatically decreased intracellular ATP levels (Fig. 2a,b). This observation indicates that OXPHOS activity is necessary for survival of glycolysis-suppressed PANC-1 cells.

Next, to assess mitochondrial morphology, we observed PANC-1 cells using transmission electron microscopy. We found that mitochondrial structure was sharper, and that mitochondrial fusion, a dynamic process, could be more clearly observed in glycolysis-suppressed PANC-1 cells (Fig. 2c, Supplementary Fig. S2a).

To investigate further mitochondrial function, we assessed mitochondrial membrane potential by JC-1 staining. Accumulation of the polymeric form of JC-1 indicates high uptake of the stain into mitochondria, which corresponds to high mitochondrial membrane potential^32^. In PANC-1 cells, glycolytic suppression increased the ratio of polymeric (red) to monomeric (green) JC-1, indicating that these cells had a high mitochondrial membrane potential (Fig. 2d). This increase was confirmed by high uptake of MitoTracker Orange, a dye that stains mitochondria in a membrane potential-dependent manner, in glycolysis-suppressed PANC-1 cells (Supplementary Fig. S2b). Because activated mitochondria generally consume more oxygen, we assumed that the oxygen consumption rate was higher in glycolysis-suppressed PANC-1 cells than in glycolysis-active cells. As expected, glycolytic suppression accelerated the oxygen consumption rate in the culture medium (Fig. 2e). In addition, we confirmed that glycolytic suppression increased the number of mitochondria (as measured by mitochondrial DNA content, *cytochrome c oxidase subunit II*), but the increase was not significant (Fig. 2f). Consistent with this, glycolysis-suppressed cells exhibited little change in fluorescence intensity of MitoTracker Green, which labels mitochondria regardless of membrane potential (Supplementary Fig. S2b). Taken together, these data imply that mitochondrial quality in PANC-1 cells was increased, but mitochondrial mass was hardly affected by glycolytic suppression.

We also confirmed that glycolytic suppression using the hexokinase inhibitor 2-DG could increase mitochondrial OXPHOS activity in PANC-1 cells. Exposure to 10 mM 2-DG significantly decreased lactate release without greatly affecting cell viability (Supplementary Fig. S3a,b). Under the glycolysis-suppressed condition using 2-DG, co-treatment of the OXPHOS inhibitor oligomycin (20 ng/mL) significantly decreased intracellular ATP levels (Supplementary Fig. S3c). This was similar to the effect of changing the sugar source in the culture medium, suggesting that PANC-1 cells can rely on mitochondrial OXPHOS for survival when glycolysis is suppressed.

### Intracellular energy metabolism is reprogrammed to mitochondrial OXPHOS by glycolytic suppression in PANC-1 cells

**Figure 3:**
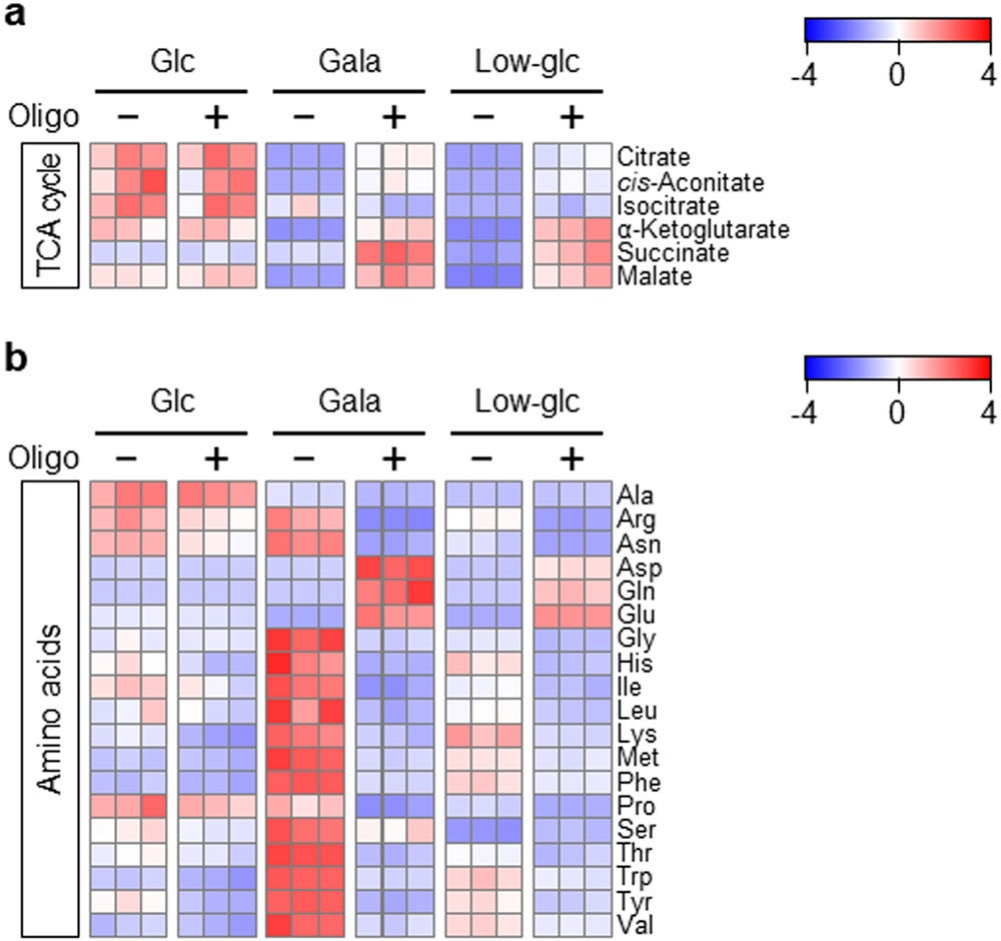
Intracellular energy metabolism is reprogrammed to mitochondrial OXPHOS by glycolytic suppression in PANC-1 cells. (**a**,**b**) PANC-1 cells were cultured in glucose (Glc), galactose (Gala), or low-glucose (Low-glc) medium with or without oligomycin (Oligo; 0.8 ng/mL) for 72 hr. The levels of metabolites extracted from the cells were measured by CE-TOFMS and normalized against the amount of total cellular protein. Metabolomic patterns of TCA cycle intermediates **(a)** and amino acids **(b)** were visualized using z-score plots and heat maps. Data represent three independent cell cultures for each condition.

As shown above, glycolytic suppression dramatically changed the metabolic profile of PANC-1 cells (Fig. 1e). To determine whether mitochondrial OXPHOS inhibition reverses these changes in glycolysis-suppressed PANC-1 cells, we measured the concentrations of each metabolite by CE-TOFMS. To assess mitochondrial function-dependent glycometabolic changes, we exposed glycolysis-suppressed PANC-1 cells to oligomycin at a concentration of 0.8 ng/mL, which did not induce detectable cell death, but did mildly inhibit mitochondrial respiration (Supplementary Fig. S4a,b). Indeed, the alterations in the metabolic profile induced by glycolytic suppression were largely reversed by exposure to oligomycin (Supplementary Fig. S4c); in particular, the decrease in the levels of TCA cycle intermediates was abolished (Fig. 3a). Based on these data, we can speculate that glycolysis-suppressed PANC-1 cells use TCA cycle intermediates to generate ATP from mitochondrial OXPHOS, and that the increase in mitochondrial respiration correlates with the reduced levels of TCA cycle intermediates. In addition, OXPHOS inhibition plus glycolytic suppression did not increase the levels of amino acids (arginine, asparagine, glycine, histidine, isoleucine, leucine, lysine, methionine, phenylalanine, serine, threonine, tryptophan, tyrosine, and valine), whereas glycolytic inhibition alone significantly increased amino acid levels (Fig. 3b). Three amino acids, glutamine, glutamate, and aspartate, exhibited patterns opposite to those of other amino acids by OXPHOS inhibition plus the glycolytic suppression in PANC-1 cells. These findings imply that dynamic changes in intracellular amino acid levels in glycolysis-suppressed PANC-1 cells are closely related to the induction of mitochondrial OXPHOS.

### Metabolic reprogramming by glycolytic suppression also occurs in other cancer cells

**Figure 4:**
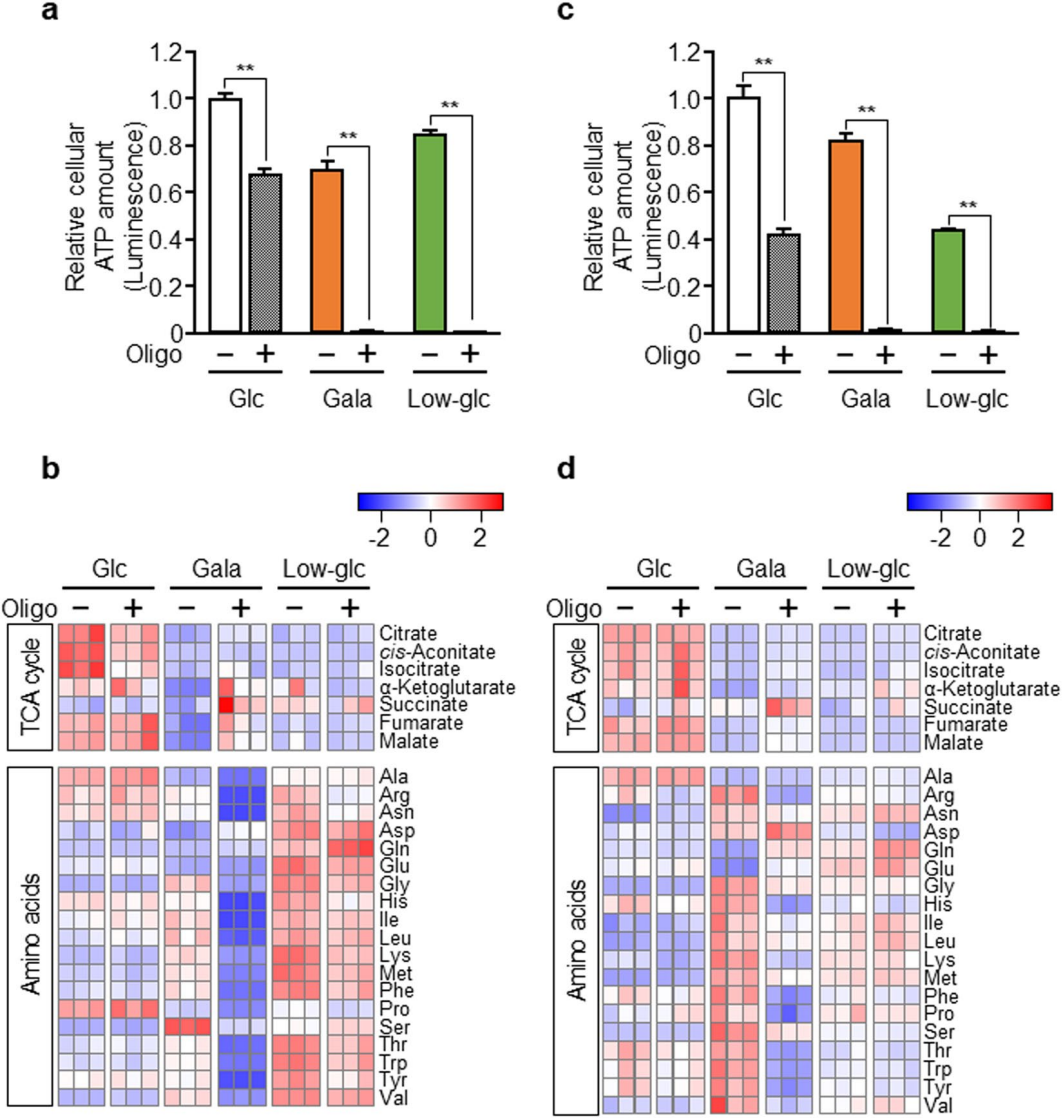
Metabolic reprogramming by glycolytic suppression also occurs in other cancer cells. (**a**,**c)** A549 **(a)** or HeLa cells **(c)** were cultured in glucose (Glc), galactose (Gala), or low-glucose (Low-glc) medium with or without oligomycin (Oligo; 20 ng/mL) for 48 hr, and then the total intracellular ATP content was quantitated. The data were normalized against the level in A549 or HeLa cells cultured in Glc medium without oligomycin. Data represent means ± SD of three independent cell cultures. **P < 0.01. (**b**,**d**) A549 (**b**) or HeLa cells **(d)** were cultured in Glc, Gala, or Low-glc medium with or without oligomycin (Oligo; 1.0 ng/mL or 0.8 ng/mL, respectively) for 72 hr, and the levels of metabolites extracted from the cells were measured by CE-TOFMS and normalized against the amount of total cellular protein. Metabolomic patterns of TCA cycle intermediates and amino acids were visualized using z-score plots and heat maps. Data represent three independent cell cultures for each condition.

The observations described above indicate that when glycolysis is inhibited, PANC-1 cells obtain energy for survival from OXPHOS by reprogramming intracellular energy metabolism. Next, we investigated whether a similar phenomenon might occur in other cancer cells. In these experiments, we attempted to obtain detailed glycometabolic profiles of reprogramming in a broad range of cancer cells. We cultured A549 human lung adenocarcinoma cells in glucose, galactose, or a low-glucose, and then assessed the contribution of OXPHOS by measuring intracellular ATP levels. OXPHOS inhibition decreased the intracellular ATP level when glycolysis was suppressed (Fig. 4a). Prior to the assessment of metabolic profiles, we confirmed that exposure to oligomycin at the concentration used in the metabolomic analysis decreased oxygen consumption but did not exert a cytotoxic effect (Supplementary Fig. S5a,b). Metabolomic analysis revealed that the levels of TCA cycle intermediates and amino acids (glycine, isoleucine, leucine, lysine, methionine, phenylalanine, serine, threonine, tryptophan, and valine) were altered in cells cultured in galactose, and that these changes were reversed by OXPHOS inhibition (Fig. 4b, Supplementary Fig. S5c). In low-glucose, the change in the level of the TCA cycle intermediates with or without oligomycin was less clear, but the levels of amino acids were dramatically shifted (Fig. 4b). These profiles were largely comparable with results obtained in PANC-1 cells. In addition, we performed similar experiments with HeLa human cervical cancer cells, and obtained results comparable with those from A549 and PANC-1 cells (Fig. 4c,d, Supplementary Fig. S6a–c). Taken together, our findings suggest that multiple types of cancer cells have the potential to reprogram intracellular energy metabolism to activate mitochondrial OXPHOS when the glycolytic pathway is suppressed.

### Influx of amino acids into the TCA cycle is important for the survival of PANC-1 cells

**Figure 5:**
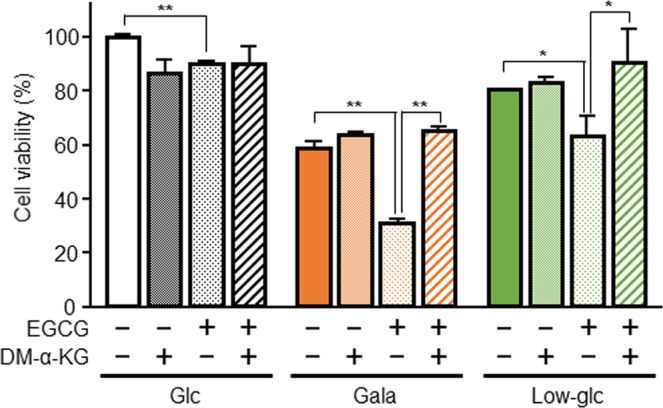
Influx of amino acids into the TCA cycle is important for the survival of PANC-1 cells. PANC-1 cells cultured in glucose (Glc), galactose (Gala), or low-glucose (Low-glc) medium for 48 hr were additionally treated with or without EGCG (25 µM) or dimethyl-α-ketoglutarate (DM-α-KG, 2 mM) for 48 hr. Cell viability was evaluated by MTT assay. The data were normalized against the level in PANC-1 cells cultured in Glc medium without EGCG or DM-α-KG. Data represent means ± SD of three independent cell cultures. *P < 0.05 and **P < 0.01.

Glutamine, glutamate, and aspartate levels decreased dramatically, a pattern opposite to those of other amino acids, in glycolysis-suppressed cancer cells. Many cancer cells rely on glutaminolysis, which converts glutamine into α-ketoglutarate (α-KG) via glutamate, for their survival^33^. To investigate whether the glutamate–α-KG axis contributes to glycolysis-suppressed PANC-1 cells’ ability to obtain survival ATP from mitochondria, we restricted the amount of intracellular α-KG using (-)-epigallocatechin gallate hydrate (EGCG), a GDH1 inhibitor, and determined the cell viability by mitochondria-dependent reduction of MTT to formazan. EGCG treatment of cells cultured in galactose or low-glucose medium decreased their viability, whereas addition of the α-KG analog dimethyl-α-KG rescued the reduction in viability (Fig. 5). These findings suggest that the influx of glutamine and glutamate into the TCA cycle in glycolysis-suppressed cells drives their mitochondrial activity, and that these two amino acids were largely consumed.

### Autophagy mediates metabolic reprogramming toward OXPHOS in glycolysis-suppressed PANC-1 cells

**Figure 6:**
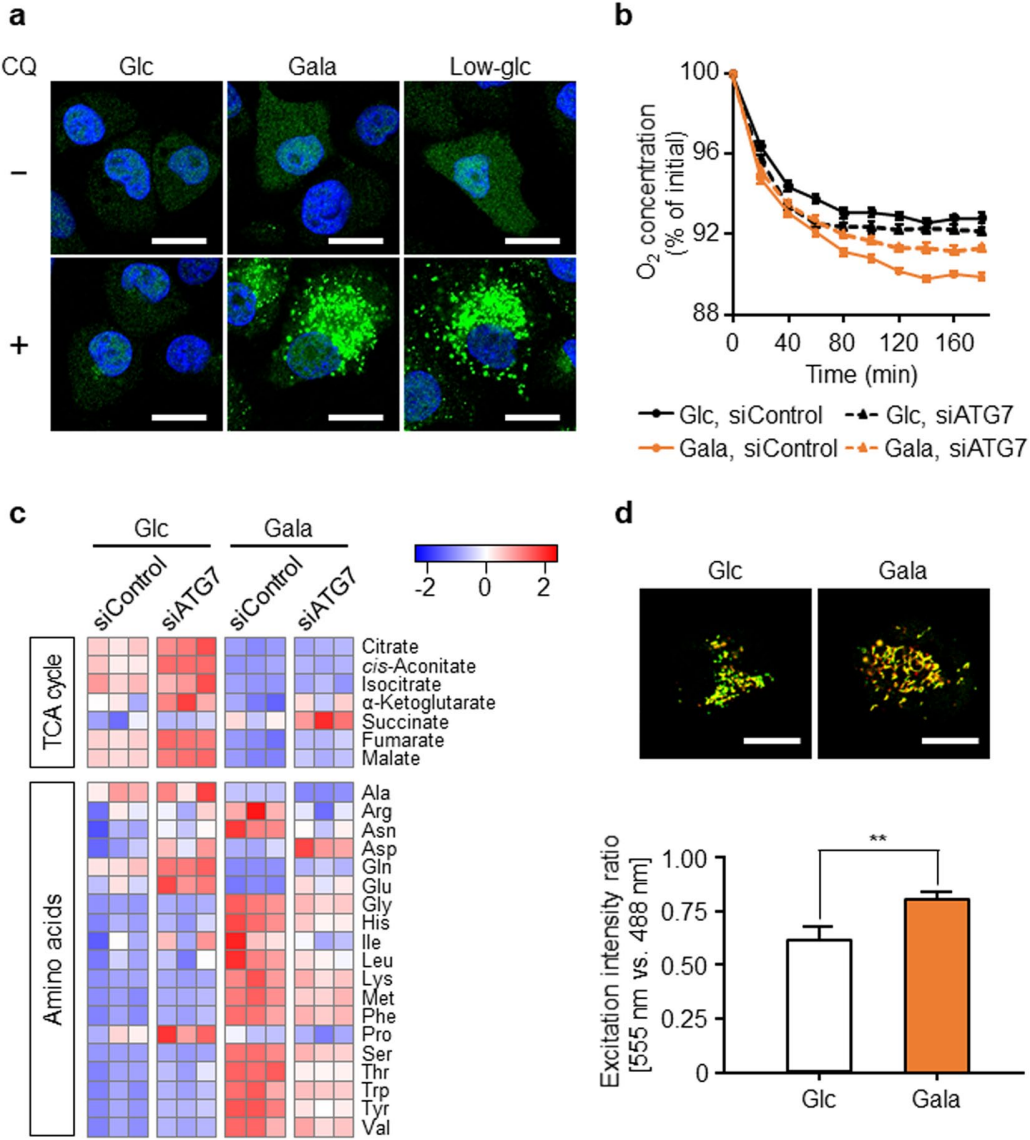
Autophagy mediates metabolic reprogramming toward OXPHOS in glycolysis-suppressed PANC-1 cells. **(a)** PANC-1 cells cultured in glucose (Glc), galactose (Gala), or low-glucose (Low-glc) medium for 72 hr were treated with or without chloroquine (CQ, 10 µM) for 48 hr, and autophagic activity was evaluated by immunostaining for endogenous LC3 (green). Nuclei were stained with TO-PRO-3 iodide (blue). Scale bars, 20 µm. **(b)** PANC-1 cells transfected with control siRNA (siControl) or siATG7 were cultured in Glc or Gala medium for 48 hr, and the oxygen concentration in the culture medium was measured over time. Data represent means ± SD of three independent cell cultures. **(c)** PANC-1 cells transfected with siControl or siATG7 were cultured in Glc or Gala medium for 48 hr, and the levels of metabolites extracted from the cells were measured by CE-TOFMS and normalized against the amount of total cellular protein. Metabolomic patterns of TCA cycle intermediates and amino acids were visualized using z-score plots and heat maps. Data represent three independent cell cultures for each condition. **(d)** PANC-1 cells cultured in Glc or Gala medium for 72 hr were transfected with mtKeima-Red plasmid, and then cultured for an additional 48 hr and observed by confocal microscopy. Representative images show merged images of excitation intensity at 488 nm (green) and 555 nm (red). Scale bars, 20 µm. The ratio of excitation intensity at 555 nm vs. 488 nm was calculated. Data represent means ± SD of three independent cell cultures. **P < 0.01.

In the cancer cells that we examined, the levels of most amino acids were increased upon replacement of sugar source, although glutamine and glutamate were consumed by the mitochondrial energy acquisition pathway. This tendency was particularly remarkable in cells cultured with galactose. To elucidate the mechanism by which these amino acids were supplied, we focused on autophagy, an intracellular self-degradation system that breaks down damaged proteins and organelles. To assess autophagic capacity in glycolysis-suppressed PANC-1 cells, we subjected cells to immunostaining to investigate the flux by monitoring turnover of the autophagosomal marker LC3 in the presence or absence of chloroquine diphosphate (CQ), an inhibitor of lysosomal degradation, at a later step of autophagy. In the presence of CQ, we detected accumulation of autophagosomes in PANC-1 cells cultured in galactose or low-glucose medium (Fig. 6a), indicating that autophagy was continuously induced by suppression of glycolysis. In addition, we could clearly visualize autophagic vacuoles in glycolysis-suppressed PANC-1 cells by transmission electron microscopy (Supplementary Fig. S2a).

Hence, we investigated the role of autophagy in glycolysis-suppressed PANC-1 cells. To suppress autophagy, we knocked down the *autophagy related 7*(*ATG7*) gene with siRNA (Supplementary Fig. S7a,b). Autophagy was suppressed under this condition, as shown by the accumulation of p62 protein, which is degraded via autophagy, and a reduction in the level of membrane-bound LC3-II (Supplementary Fig. S7b). Suppression of autophagy abolished the increase in oxygen consumption rate in PANC-1 cells cultured in galactose (Fig. 6b), but had little effect on cells cultured in glucose, indicating that autophagy positively regulates mitochondrial OXPHOS in glycolysis-suppressed cells. Next, we investigated whether suppression of autophagy alters the metabolic profile in glycolysis-suppressed PANC-1 cells, as OXPHOS inhibition did. The reductions in the levels of TCA cycle intermediates and the elevations in the levels of amino acids (arginine, asparagine, glycine, histidine, isoleucine, leucine, lysine, methionine, phenylalanine, serine, threonine, tryptophan, tyrosine, and valine) tended to be reversed by knockdown of ATG7 (Fig. 6c, Supplementary Fig. S7c). These data suggest that autophagy supplies the amino acids necessary for driving mitochondrial OXPHOS in glycolysis-suppressed cells.

Furthermore, to investigate the role of autophagy-mediated mitochondrial turnover in the maintenance of functional homeostasis in glycolysis-suppressed cells, we focused on mitophagy, a form of selective autophagy that degrades damaged mitochondria. To evaluate the mitophagic capacity in glycolysis-suppressed PANC-1 cells, we have transfected cells with an expression vector encoding mitochondrially targeted Keima-Red (mtKeima-Red)^34^. As shown in Fig. 6d, PANC-1 cells cultured with galactose exhibited a higher excitation intensity ratio [555 nm (red) vs. 488 nm (green)] than cells cultured with glucose, indicating that higher levels of mitochondria were localized in acidic environments. Thus, mitophagy was induced in glycolysis-suppressed cells, implying that mitophagy contributes to the increase in mitochondrial function. Collectively, these results suggest that autophagic process mediates metabolic reprogramming toward mitochondrial OXPHOS.

**Figure 7:**
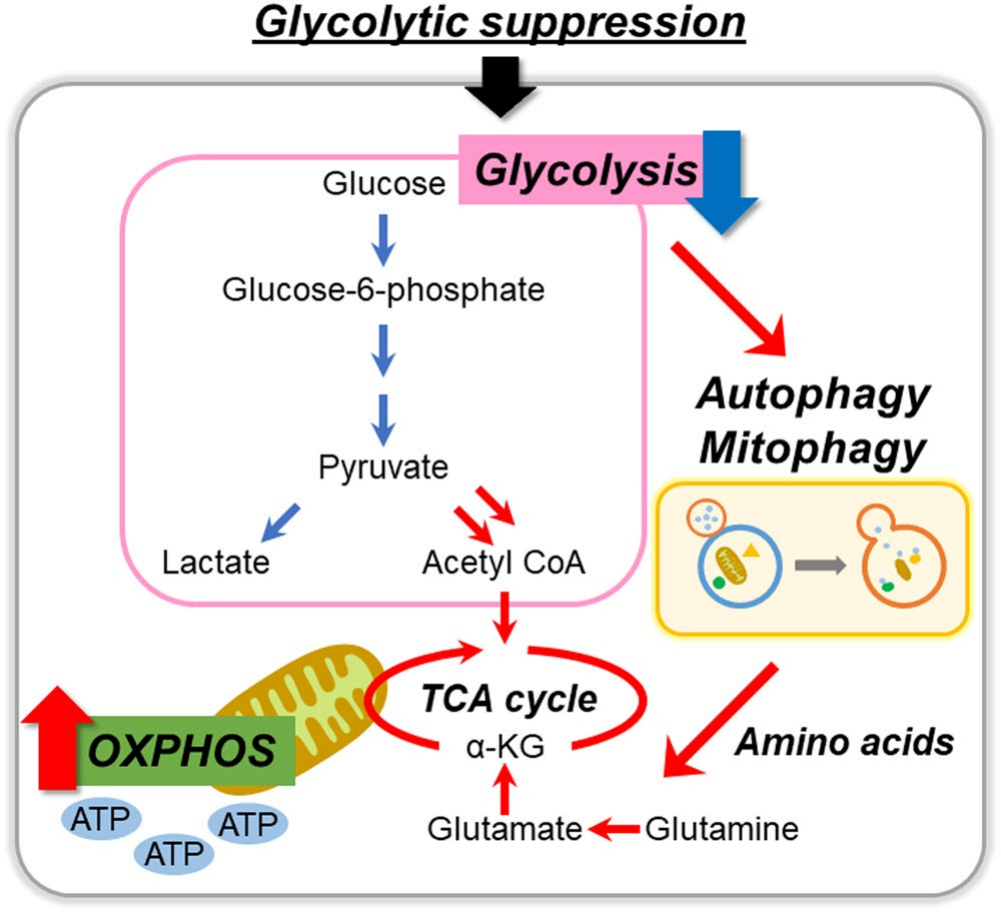
Overview of study findings. Glycolytic suppression reprograms intracellular energy metabolism toward mitochondrial OXPHOS in multiple types of cancer cells. Autophagic process mediates this metabolic reprogramming. In this process, the supply of specific amino acids and quality control of mitochondria by mitophagy play important roles.

An overview of our findings is provided in Fig. 7.

## Discussion

In this study, we demonstrated that glycolytic suppression in PANC-1 pancreatic cancer cells and two other solid tumor cell lines, A549 and HeLa, upregulated mitochondrial function and reprogrammed glycometabolism to OXPHOS. The reduction in the levels of TCA cycle intermediates caused by glycolytic suppression were reversed by OXPHOS inhibition. In addition to the results of metabolome analysis, the levels of TCA cycle enzymes such as SUCLA2 and MDH2, as well as OXPHOS enzymes, were increased by glycolytic suppression (Supplementary Fig. S1a). SUCLA2 converts succinyl-CoA to succinate and MDH2 converts malate to oxaloacetate. Therefore, the elevated levels of these enzymes suggest that the TCA cycle was activated by glycolytic suppression. In addition, the influx of glutamine and glutamate into the TCA cycle contributed to the survival of glycolysis-suppressed PANC-1 cells. In proliferating tumor cells, glutamine is used as a carbon source for TCA cycle intermediates and a nitrogen source for nonessential amino acids, nucleotides, and hexosamine^35^. Glutamine is converted to glutamate via glutaminase, and then three enzymes convert glutamate to α-KG. One of these enzymes is GDH and the others are transaminases, GOT and glutamate pyruvate transaminase^36^. In human cancer cells, including lung cancer and breast cancer cells, the influx of glutamate into the TCA cycle is mainly mediated by GDH1^37^. We found that glycolysis-suppressed PANC-1 cells exhibited high sensitivity to GDH1 inhibitor (Fig. 5), suggesting that the influx of glutamate into the TCA cycle through GDH1 is important for acquiring energy from mitochondria. In addition, glutamine-derived aspartate is converted by GOT1 into oxaloacetate, an intermediate of the TCA cycle, in pancreatic ductal adenocarcinoma^38^. Consistent with this observation, we showed that GOT1 was upregulated in glycolysis-suppressed PANC-1 cells (Supplementary Fig. S1a). We also found that glycolytic suppression significantly increased the levels of amino acids, although not in the case of aspartate (Figs. 1e, 3b and 4b). These results indicated that aspartate flows into the TCA cycle via conversion to oxaloacetate in glycolysis-suppressed PANC-1 and A549 cells. Further investigation of the biosynthesis and utilization of these amino acids in glycolysis-suppressed cells is required.

Here, we showed that glycolytic suppression induced autophagy as a mechanism of supplying amino acids (Fig. 6a). In particular, we showed that the increase in amino acid levels caused by glycolytic suppression was reversed by suppressing autophagy, although three amino acids, glutamine, glutamate, and aspartate, exhibited opposite behaviors (Fig. 6c). Other group have also observed significant accumulation of amino acids, except for glutamine, in colon and stomach tumors, implying that the supply of amino acids is maintained by autophagic degradation of proteins, and that glutamine is used in glutaminolysis^17^. Therefore, our results support both of these ideas. Our previous study showed that autophagy is important for the survival of leukemia cells when the glycolytic pathway is suppressed^30^. Moreover, exposure to anti-cancer reagents (dexamethasone or imatinib) suppresses glycolysis and subsequently induces autophagy in leukemia cells^25,39^. Autophagy is critical for the maintenance of cellular homeostasis and cell survival under stressful conditions, including nutrient starvation^40^. In addition, the mammalian target of rapamycin complex 1 (mTORC1) is a master regulator of cellular metabolism that regulates autophagy, and amino acids are crucial regulators of mTORC1^41^. Recent work in yeast showed that glutamine bound the Pib2 complex, which plays a role in TORC1 activation, and that glutamine promotes formation of the Pib2–TORC1 complex, thereby regulating regulation of autophagy^42^. Furthermore, glutaminolysis via GDH is required for lysosomal translocation and activation of mTORC1 by glutamine and leucine, contributing to regulation of autophagy in cancer cells^43,44^. In this study, our results suggest that a reduction of glutamine or α-ketoglutarate associated with the use of these metabolites, which are required for the enhancement of the TCA cycle, can drives autophagy for the nutrient supply.

In this study, we found that mitochondrial function is upregulated by glycolytic suppression in the cancer cells we examined (Figs. 2 and 4a,c). Cellular energetic states affect the mitochondrial fusion/fission machinery, and mitochondrial fusion contributes to OXPHOS activity through the regulation of mitochondrial DNA content^45^. When HeLa cells are cultured in galactose medium, the mitochondria become elongated^31^. Consistent with this, we observed that glycolytic suppression dramatically changed mitochondrial morphology; in particular, mitochondrial fusion was more active in cells cultured in galactose and low-glucose medium. These results suggest that mitochondrial dynamic structure can be remodeled to accommodate a change in OXPHOS activity. On the other hand, we showed that glycolytic suppression increased mitochondrial membrane potential (Fig. 2d, Supplementary Fig. S2b), but did not have a significant effect on mitochondrial mass (Fig. 2f, Supplementary Fig. S2b). Therefore, we suggest that the increase in mitochondrial OXPHOS activity caused by glycolytic suppression is due to an increase in mitochondrial quality, not quantity.

Several studies have shown that mitochondrial remodeling to upregulate activity is controlled by mitophagy. Mitophagy regulates mitochondrial number to match metabolic demand and contributes to maintenance of mitochondrial quality^46,47^. When OXPHOS activity is high, the small GTPase Rheb is recruited to the mitochondrial outer membrane, promoting mitophagy to maintain optimal energy production from mitochondria^48^. Furthermore, activation of AMP-activated protein kinase (AMPK) by energy stress can activate mitophagy via phosphorylation of ULK1 or ULK2, which are mammalian protein kinases required for induction of autophagy, thus promoting mitochondrial homeostasis and cell survival^49^. Taken together, these observations suggest that mitophagy contributes to metabolic reprogramming of OXPHOS by maintaining mitochondrial quality and energy acquisition when the glycolytic pathway is suppressed.

Consistent with our previous report^30^, our findings in this study also suggest that autophagy acts as a positive regulator of mitochondrial OXPHOS when glycolysis is suppressed. In addition, glycolytic suppression induced mitophagy (Fig. 6d), suggesting that mitophagy contributes to maintenance of mitochondrial function. Therefore, targeting autophagy and/or mitophagy represents a potential strategy for regulating OXPHOS activity. Clinical studies of autophagy inhibitors have been conducted; e.g., CQ and hydroxychloroquine are used in combination therapies along with anti-cancer agents^50^. To date, however, it remains unclear how combination therapy with anti-cancer agents and autophagy inhibitors exerts its effects. Our findings suggest that these autophagic inhibitors may diminish the increase in OXPHOS activity induced by anti-cancer agents. However, autophagy is important for the maintenance of cellular homeostasis, not only in cancer cells but also in normal cells^51^, and bulk autophagic inhibition may affect normal cells. Therefore, cancer-specific regulation of mitophagy represents a promising candidate for cancer therapy targeting intracellular energy metabolism. In particular, the pathway mediated by PTEN-induced putative kinase 1 (PINK1) and the E3 ubiquitin ligase Parkin has been well studied as a trigger of mitophagy^52^. Parkin is selectively recruited to depolarized mitochondria via stabilization of PINK1 at the outer mitochondrial membrane, resulting in Parkin-induced mitophagy^53,54^. However, Parkin expression is downregulated in most types of cancer cells^55,56^. Recent work showed that Ariadne RING-between-RING (RBR) E3 ubiquitin protein ligase 1 (ARIH1), which like Parkin belongs to the RBR E3 ligase subfamily, controls mitophagy in human lung cancer cells in a PINK1-dependent manner^57^. Although the regulation of mitophagy of cancer cells remains largely unclear, inhibiting specific factors like ARIH1 in lung cancer cells should improve the efficacy and safety of anti-cancer treatment. Glycolytic suppression plus OXPHOS inhibition represents a potentially efficient strategy for targeting cancer metabolism. Indeed, suppressing glycolysis by inhibiting or knocking down LDHA in combination with phenformin to inhibit OXPHOS decreases pancreatic tumor cell growth *in vitro* and *in vivo*^27^. However, because healthy cells rely on OXPHOS, inhibition of this process could have unwanted effects. Therefore, we are convinced that targeting the cancer-specific regulatory mechanism of mitophagy is a promising strategy for cancer treatment.

In this study, glycolytic suppression resulted in reprogramming of glycometabolism toward mitochondrial OXPHOS in multiple solid tumors, consistent with several recent reports^27–29^. In addition, we showed that autophagy contributes to the amino acid supply and maintenance of mitochondrial function in glycolysis-suppressed PANC-1 cells. It remains unclear whether cancer cells sense glycolytic suppression and send signals to the autophagy-dependent mitochondrial OXPHOS pathway. A recent report indicated that theoretical modeling combining gene expression profiles with metabolic pathways is an important strategy for comprehensive characterization of cancer metabolism^58^. Thus, further studies combining multiple forms of omics analyses could provide a deeper understanding of the regulatory mechanisms of cancer energy reprogramming.

In conclusion, we have shown that intracellular glycometabolism is reprogrammed to mitochondrial OXPHOS to promote survival when the glycolytic pathway is suppressed using multiple cancer cells of solid tumors. Moreover, specific amino acids such as glutamine and glutamate are important for metabolic reprogramming. Furthermore, glycolytic suppression induced autophagy, boosting the amino acid supply, and also activated mitophagy. These findings suggest that intracellular energy metabolism is reprogrammed toward mitochondrial OXPHOS, and that the supply of specific amino acids by autophagy and quality control of mitochondria by mitophagy play important roles in this process. Our findings help to explain some of the mechanisms underlying the efficacy of combination therapies that affect the dynamics of intracellular energy metabolism. We firmly believe that comprehensive understanding of cancer energy metabolism is necessary for confronting many aspects and varieties of cancer cells.

## Materials and Methods

### Reagents

CQ, purchased from Sigma-Aldrich (St. Louis, MO, USA), was dissolved in MilliQ water. Dimethyl-α-KG was obtained from Tokyo Kasei (Tokyo, Japan). EGCG (Tokyo Kasei), rotenone (Wako, Osaka, Japan), and oligomycin (Sigma) were dissolved in dimethyl sulfoxide (DMSO). The final DMSO concentration in cell culture did not exceed 0.5% (v/v).

### Cell culture

All cells were purchased from RIKEN Cell Bank (Tsukuba, Japan). PANC-1 and A549 cells were maintained in RPMI-1640 medium (Nacalai Tesque, Kyoto, Japan), and HeLa cells in MEM (Nacalai Tesque), supplemented with 10% fetal bovine serum (Life Technologies, Grand Island, NY, USA) plus antibiotics (Nacalai Tesque). Cells were cultured at 37 °C in a humidified atmosphere of 5% CO_2_ in air. In each experiment, PANC-1 and A549 cells were cultured in RPMI-1640 containing glucose (2 g/L), galactose (2 g/L), or low-glucose (0.2 g/L). HeLa cells were cultured in MEM containing glucose (1 g/L), galactose (1 g/L), or low-glucose (0.1 g/L).

### Measurement of cellular lactate release

The amount of cellular lactate release was measured as described previously^30^. Briefly, the supernatant from cultured cells was de-proteinized with perchloric acid and neutralized with potassium hydroxide. The supernatant was mixed with nicotinamide adenine dinucleotide and glutamate pyruvate transaminase (Roche, Mannheim, Germany). The enzymatic reaction was started by adding lactate dehydrogenase (Wako) to each sample and incubating at 37 °C for 30 min. Absorbance was measured at a wavelength of 340 nm.

### Flow cytometric detection of proliferating and dead cells

To analyze cell proliferation rate, PANC-1 cells were incubated for 15 min with 5 µM CFSE (Dojin, Kumamoto, Japan) dissolved in FBS-free RPMI-1640. After dye loading, cells were washed and cultured for 4 days. To distinguish dead cells from live cells, cultured cells were collected and stained with PI (SONY, Tokyo, Japan). Data acquisition was performed on an EC800 cell analyzer (SONY).

### Metabolome analysis

Extraction of cellular metabolites and analysis were performed as previously described^17,59^. For extraction of metabolites, cultured cells were washed twice with 5% mannitol solution, and then treated with methanol containing internal standards (Human Metabolome Technologies, Tsuruoka, Japan). After adding MilliQ water and chloroform, the extract was centrifuged and the extract was filtrated. The filtrate was concentrated by centrifugation and dissolved in 25 µL MilliQ water before measurement. The concentrations of metabolites in samples were measured by CE-TOFMS (Agilent Technologies, Santa Clara, CA, USA).

### Measurement of intracellular ATP content

Intracellular ATP concentration was assessed using the CellTiter-Glo Luminescent Cell Viability Assay (Promega, Madison, WI, USA).

### Transmission electron microscopic analysis

To observe intracellular structure, PANC-1 cells grown on culture dishes were fixed with pre-warmed glutaraldehyde at a final concentration of 2.5% at 37 °C for 15 min, and then placed at 4 °C for 24 hr. The samples were washed with 0.1 M phosphate buffer (pH 7.2), post-fixed with 1% osmium tetroxide, dehydrated with a graded series of ethanol, and embedded in an epoxy resin^60^. Ultrathin sections of 70-nm thickness were cut, stained with uranyl acetate and lead citrate, and observed on a JEM-1400 electron microscope (JEOL, Tokyo, Japan) at 4,000–10,000 × nominal magnification^61^.

### Evaluation of mitochondrial membrane potential

PANC-1 cells were incubated for 30 min with 500 nM JC-1 (Life Technologies) dissolved in RPMI-1640. JC-1 monomers can be detected using excitation and emission wavelengths of 490 and 530 nm, and JC-1 polymers at 525 and 590 nm, respectively. After loading of JC-1, images were obtained using a Carl Zeiss LSM700 laser scanning confocal microscope (Prenzlauer, Berlin, Germany).

### Measurement of cellular oxygen consumption rate

Oxygen consumption rate in cells was measured using a fluorescent oxygen probe, PreSens Sensor Dish Reader (Regensburg, Germany). Oxygen tension was monitored continuously every minute, and the concentration at time 0 was defined as 100%.

### Analysis of mitochondrial DNA content

Mitochondrial DNA content of PANC-1 cells was quantified as described previously^39^. Briefly, total cellular DNA of mitochondria and nuclei was isolated using the NucleoSpin Tissue kit (Macherey Nagel, Düren, Germany). Total DNA was mixed with THUNDERBIRD quantitative real-time PCR mix (TOYOBO, Osaka, Japan). The mixture was subjected to quantitative real-time PCR using a LightCycler 96 Real-Time PCR System (Roche). Primers were designed accordingly: mitochondrial *cytochrome c oxidase subunit II* forward, 5′-CCC CAC ATT AGG CTT AAA AAC AGA T-3′; reverse, 5′-TAT ACC CCC GGT CGT GTA GCG GT-3′; *β-actin* forward, 5′-TTC AAC ACC CCA GCC ATG TAC G-3′; reverse, 5′-GTG GTG GTG AAG CTG TAG CC-3′. Cycling conditions were as follows: 95 °C for 60 s, followed by 40 cycles at 95 °C for 10 s and 60 °C for 60 s. Relative amounts of mitochondrial DNA in cells were calculated after normalization against nuclear *β-actin* DNA.

### MTT cell viability assay

For MTT assays, PANC-1 cells were incubated with 0.5 mg/ml MTT (Dojin) for 2 hr. After the supernatant was removed, formazan produced by the mitochondria of viable cells was extracted from cells with 200 μL of DMSO. The amount of MTT-formazan was measured by monitoring absorbance at 540 nm.

### Immunostaining

Cells were fixed in PBS containing 4% formaldehyde, permeabilized in PBS containing 0.05% Triton X-100, immunostained with a rabbit anti-LC3B primary antibody (Cell Signaling Technology, Beverly, MA, USA), and labeled with a secondary antibody conjugated to an Alexa Fluor dye (Life Technologies). Nuclei were stained with TO-PRO-3 iodide (Life Technologies). Fluorescence was detected on a Carl Zeiss LSM700 laser scanning confocal microscope.

### RNA interference targeting ATG7

PANC-1 cells were transiently transfected with ATG7-targeting and control siRNAs (Sigma) (siATG7 and siControl, respectively) using Lipofectamine 2000 (Life Technologies). The sequences of the two oligonucleotide strands of siATG7 duplex were as follows: sense, 5′-GCC AGA GGA UUC AAC AUG ATT-3′; antisense, 5′-UCA UGU UGA AUC CUC UGG CTT-3′.

### Plasmid construction of mtKeima-Red, transfection, and live cell imaging

The mitochondria-targeting amino acid sequence MLSLRQSIRFFKPATRTLCSSR, derived from cytochrome oxidase subunit IV, was inserted into plasmid phmKeima-Red-MCL (MBL, Nagoya, Japan). The resultant mtKeima-Red DNA was introduced into PANC-1 cells using Lipofectamine 2000. 48 hr after transfection, cell images were obtained using a Carl Zeiss LSM700 laser scanning confocal microscope. mtKeima-Red has an excitation spectrum that varies according to pH and an emission spectrum peak at 620 nm. In a neutral environment, the excitation wavelength of 440 nm is predominant, whereas in an acidic environment, excitation at 586 nm is predominant^34^. In mitophagy, mitochondria are degraded by the autophagy–lysosome pathway. A subset of mitochondria undergoing mitophagy localize in the lysosome, an acidic vesicle, and consequently have a high ratio of mtKeima-Red excitation intensity at 586 vs. 440 nm.

### Statistical analysis

All data are expressed as means ± SD of at least three independent experiments unless indicated. Statistical analysis was performed using Student’s t test or an analysis of variance followed by the Bonferroni test, where applicable.

## Supplementary information

Supplementary Information
