# Supplementary material for: Glycolytic suppression dramatically changes the intracellular metabolic profile of multiple cancer cell lines in a mitochondrial metabolism-dependent manner
Source: Sci Rep. 2019 Dec 10;9:18699. doi: 10.1038/s41598-019-55296-3 (PMC6904735; doi:10.1038/s41598-019-55296-3)
Supplement: Supplementary file 1 — Supplementary Information [file 41598_2019_55296_MOESM1_ESM.pdf]

Title page

Glycolytic suppression dramatically changes the intracellular metabolic profile of multiple cancer cell lines in a mitochondrial metabolism-dependent manner

Reika Shiratori[1], Kenta Furuichi[1], Masashi Yamaguchi[2], Natsumi Miyazaki[1], Haruna Aoki[1], Hiroji Chibana[2], Kousei Ito[1], Shigeki Aoki[1]*

[1]Laboratory of Biopharmaceutics, Graduate School of Pharmaceutical Sciences, Chiba University, Inohana 1-8-1, Chuo-ku, Chiba-city, Chiba 260-8675, Japan.

[2]Medical Mycology Research Center, Chiba University, Inohana 1-8-1, Chuo-ku, Chiba-city, Chiba 260-8673, Japan.

*Corresponding author: Shigeki Aoki

Laboratory of Biopharmaceutics, Graduate School of Pharmaceutical Sciences, Chiba University, Inohana 1-8-1, Chuo-ku, Chiba-city, Chiba 260-8675, Japan.

Tel: +81 43 226 2888, Fax: +81 43 226 2888, E-mail: aokishigeki@chiba-u.jp

Supplementary Figures

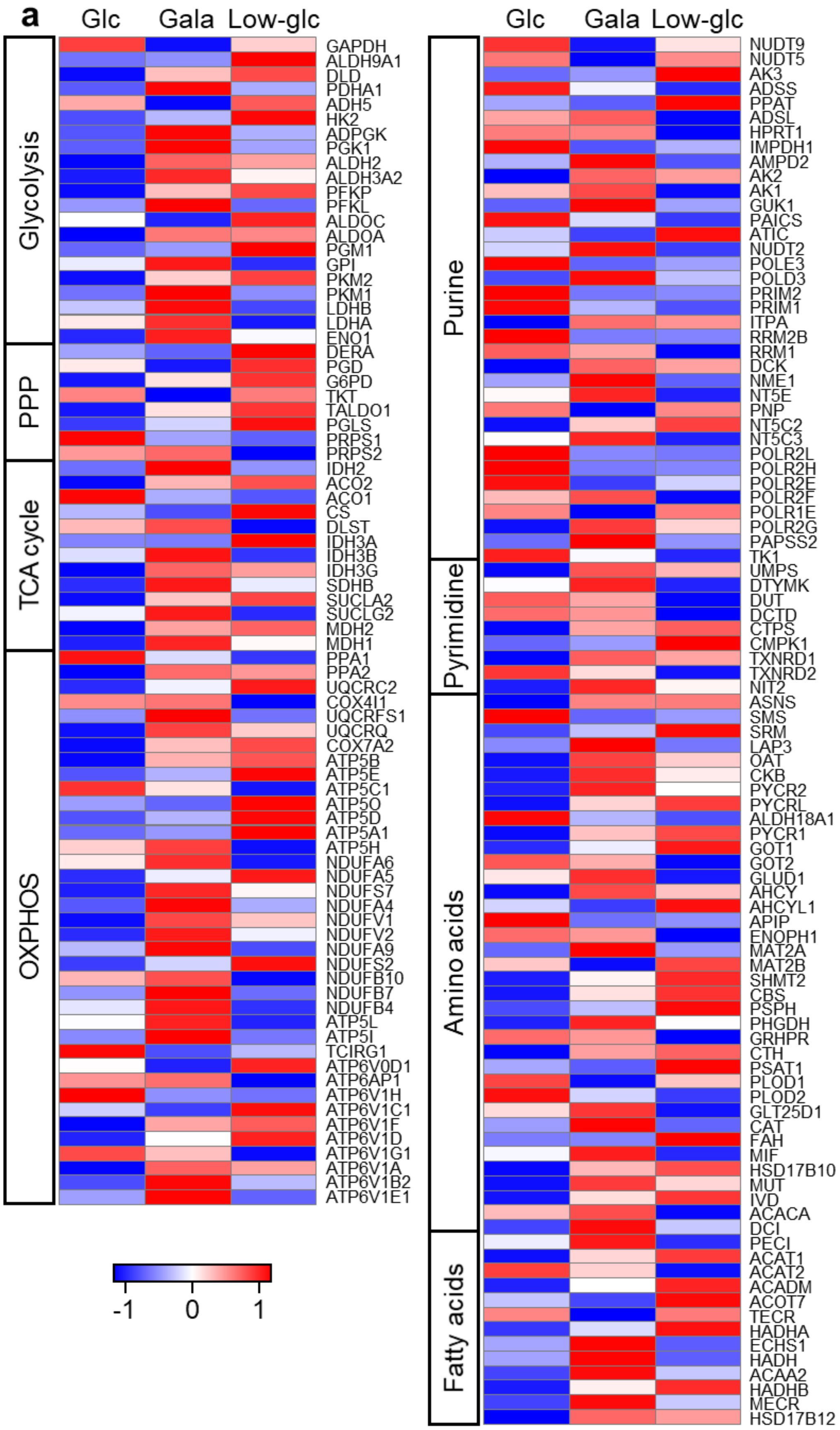

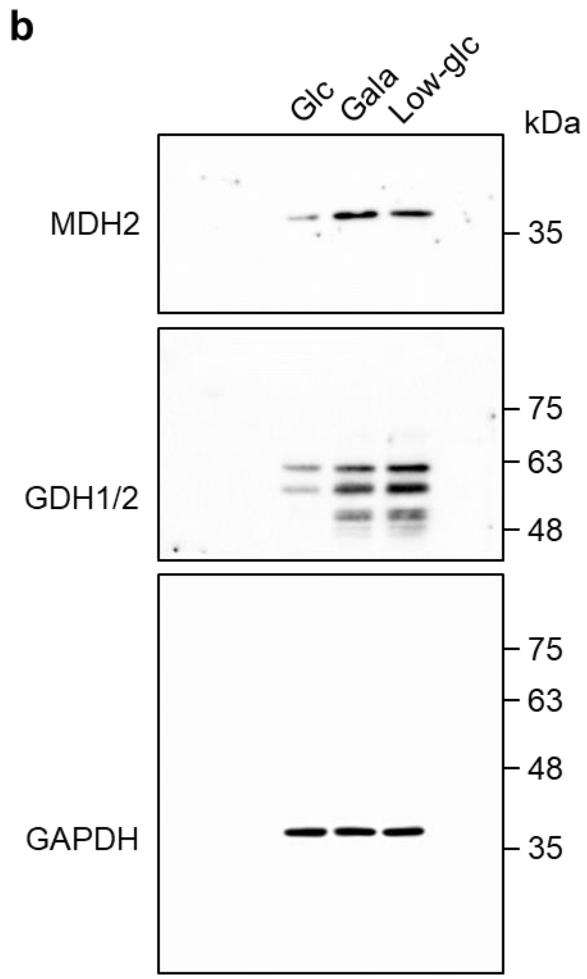

Supplementary Fig. S1. Glycolytic suppression dynamically changes the expression levels of enzymes in PANC-1 cells. (a) Levels of enzymes extracted from PANC-1 cells cultured in glucose (Glc), galactose (Gala), or low-glucose (Low-glc) medium for 48 hr were measured by LC-MS/MS and normalized against the amount of total cellular protein. Proteomic patterns were visualized using z-score plots and heat maps. **(b)** Cell lysates prepared from PANC-1 cells cultured in Glc, Gala, or Low-glc medium for 48 hr were subjected to western blotting with anti-MDH2, anti-GDH1/2, and anti-GAPDH antibodies.

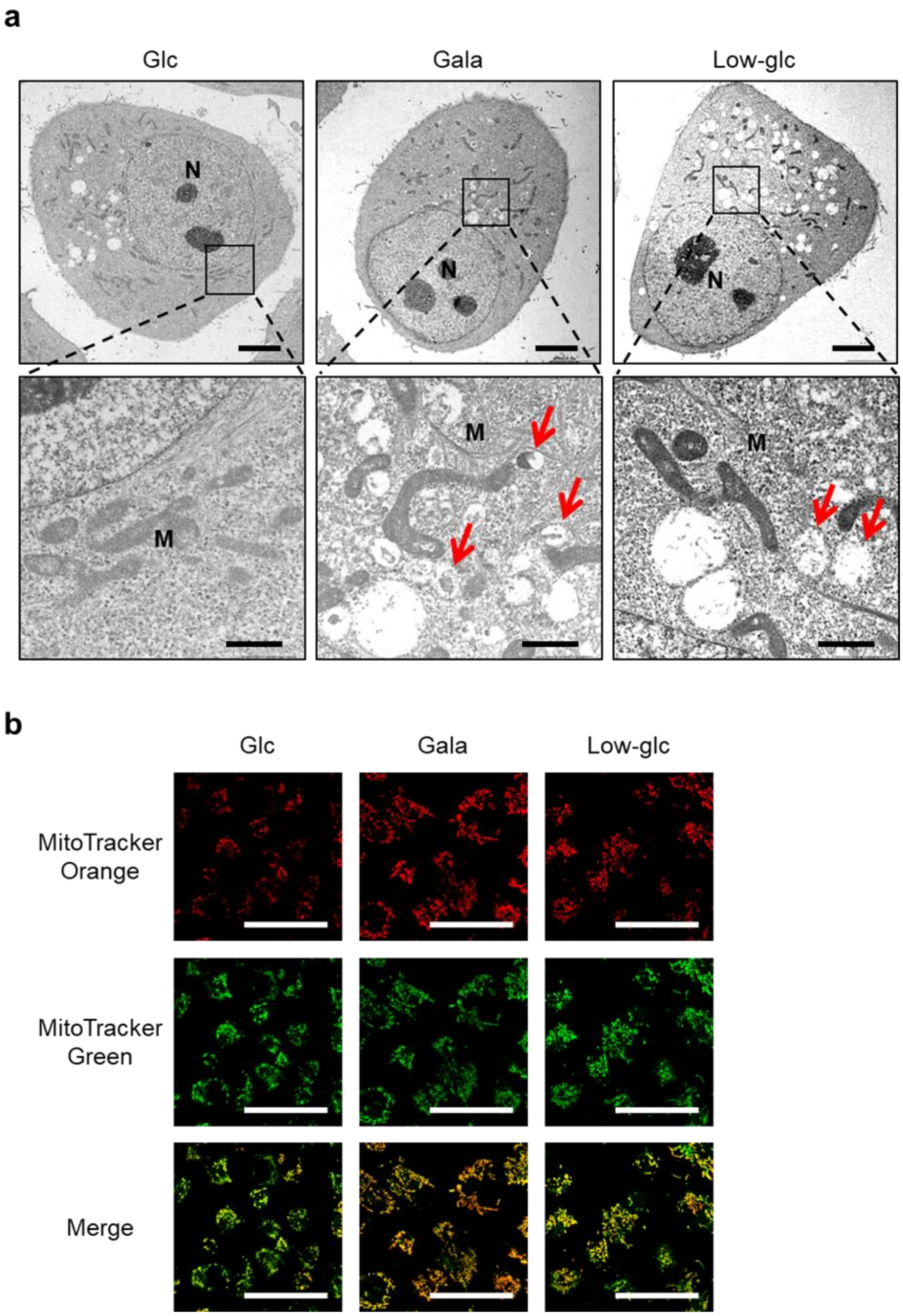
a
Glc
Gala
Low-glc
N
N
N
M
M
M
b
Glc
Gala
Low-glc
MitoTracker Orange
MitoTracker Green
Merge

Supplementary Fig. S2. Glycolytic suppression changes mitochondrial morphology and increases the formation of autophagosomes. (a) Enlarged images of PANC-1 cells presented in Fig. 2C. “N” indicates the nucleus, “M” indicates mitochondria, and red arrows indicate autophagic bodies. **(b)** PANC-1 cells cultured in glucose (Glc), galactose (Gala), or low-glucose (Low-glc) medium for 48 hr were stained with MitoTracker Orange (red) and MitoTracker Green (green) and observed by confocal microscopy. Scale bars, 50 μm.

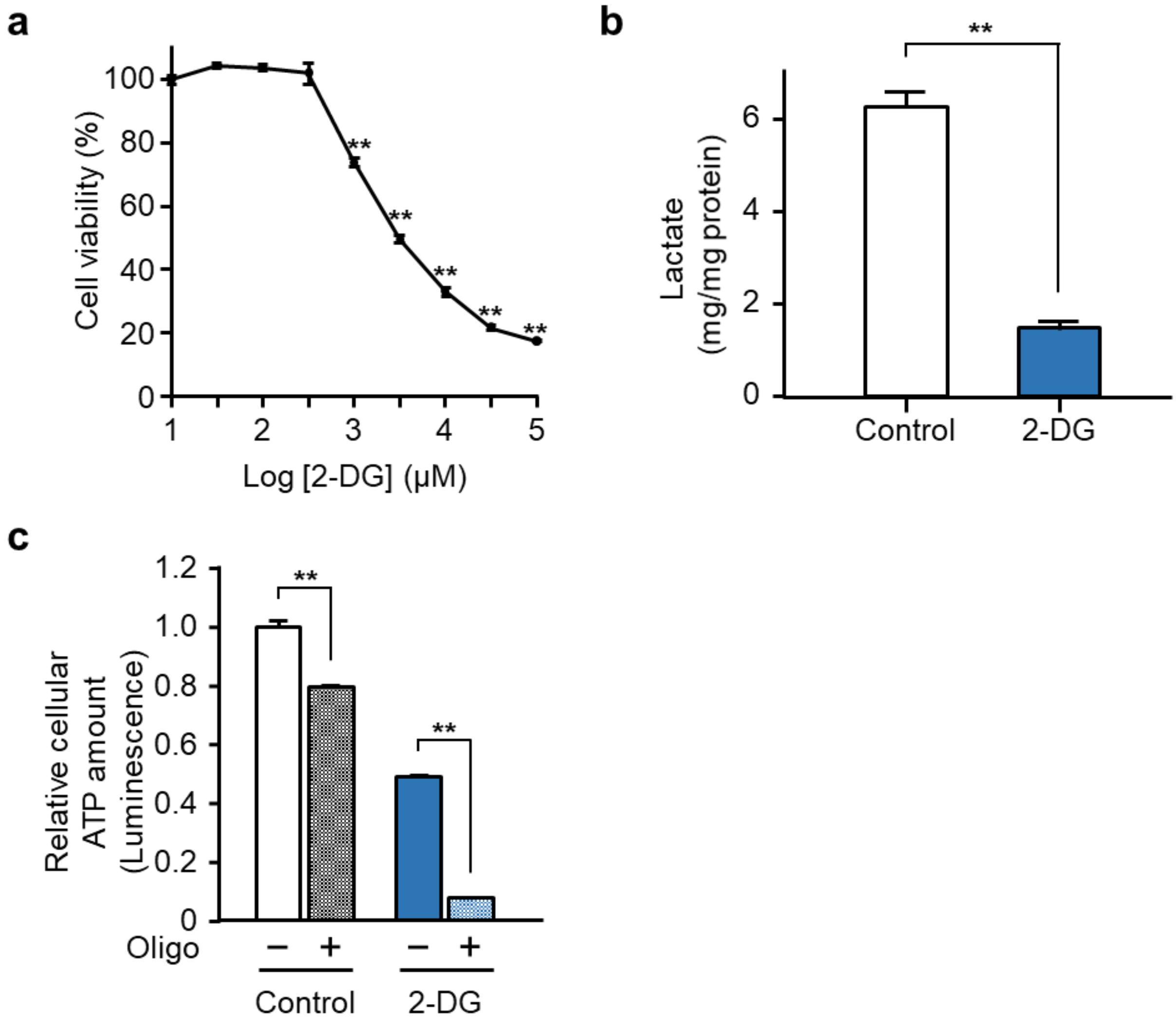

Supplementary Fig. S3. Glycolytic suppression by 2-deoxyglucose (2-DG) also upregulates mitochondrial function. (a) PANC-1 cells were treated with different concentrations of 2-DG (0–100 mM) for 72 hr, and then cell viability was evaluated by MTT assay. Data represent means ± SD of three independent cell cultures. ** $P < 0.01$, compared with cells not treated with 2-DG. **(b)** PANC-1 cells were treated with 2-DG (10 mM) for 48 hr. The amount of lactate released into the medium from the cells for the last 24 hr was measured. Data represent means ± SD of three independent cell cultures. ** $P < 0.01$. **(c)** PANC-1 cells were treated with 2-DG (10 mM) with or without oligomycin (Oligo; 20 ng/mL) for 48 hr, and then intracellular ATP content was quantitated. Data were normalized against the level in PANC-1 cells not treated with 2-DG or oligomycin. Data represent means ± SD of three independent cell cultures. ** $P < 0.01$.

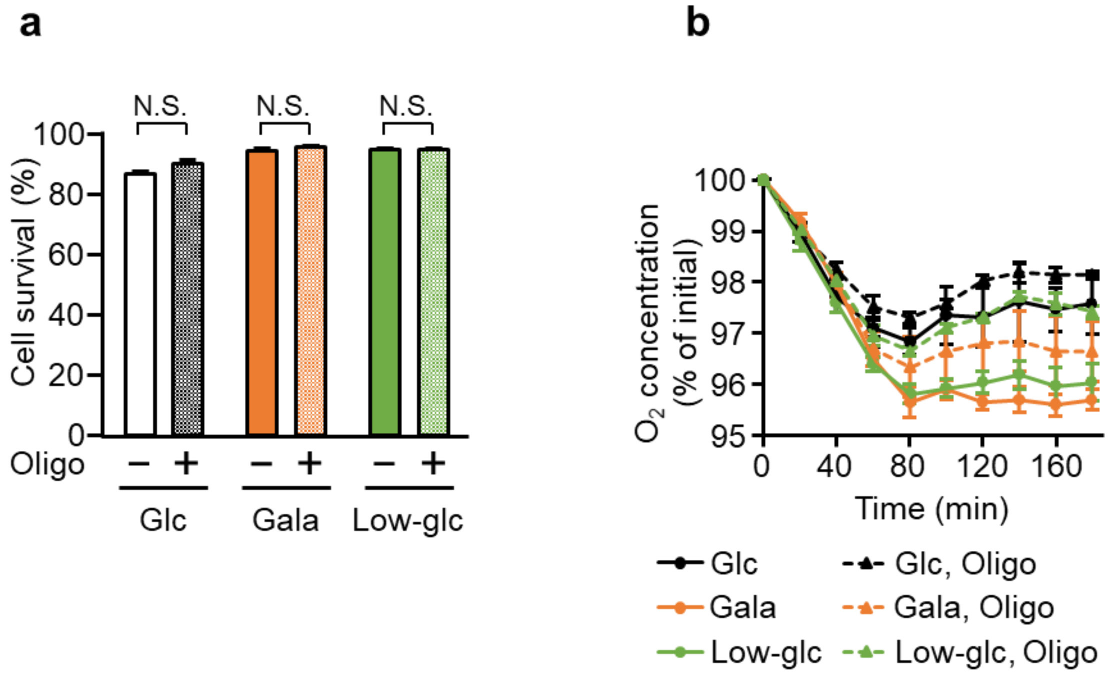
a
b
N.S.
N.S.
N.S.
Cell survival (%)
100
80
60
40
20
0
Oligo
−
+
−
+
−
+
Glc
Gala
Low-glc
O_2 concentration (% of initial)
100
99
98
97
96
95
0
40
80
120
160
Time (min)
Glc
Glc, Oligo
Gala
Gala, Oligo
Low-glc
Low-glc, Oligo

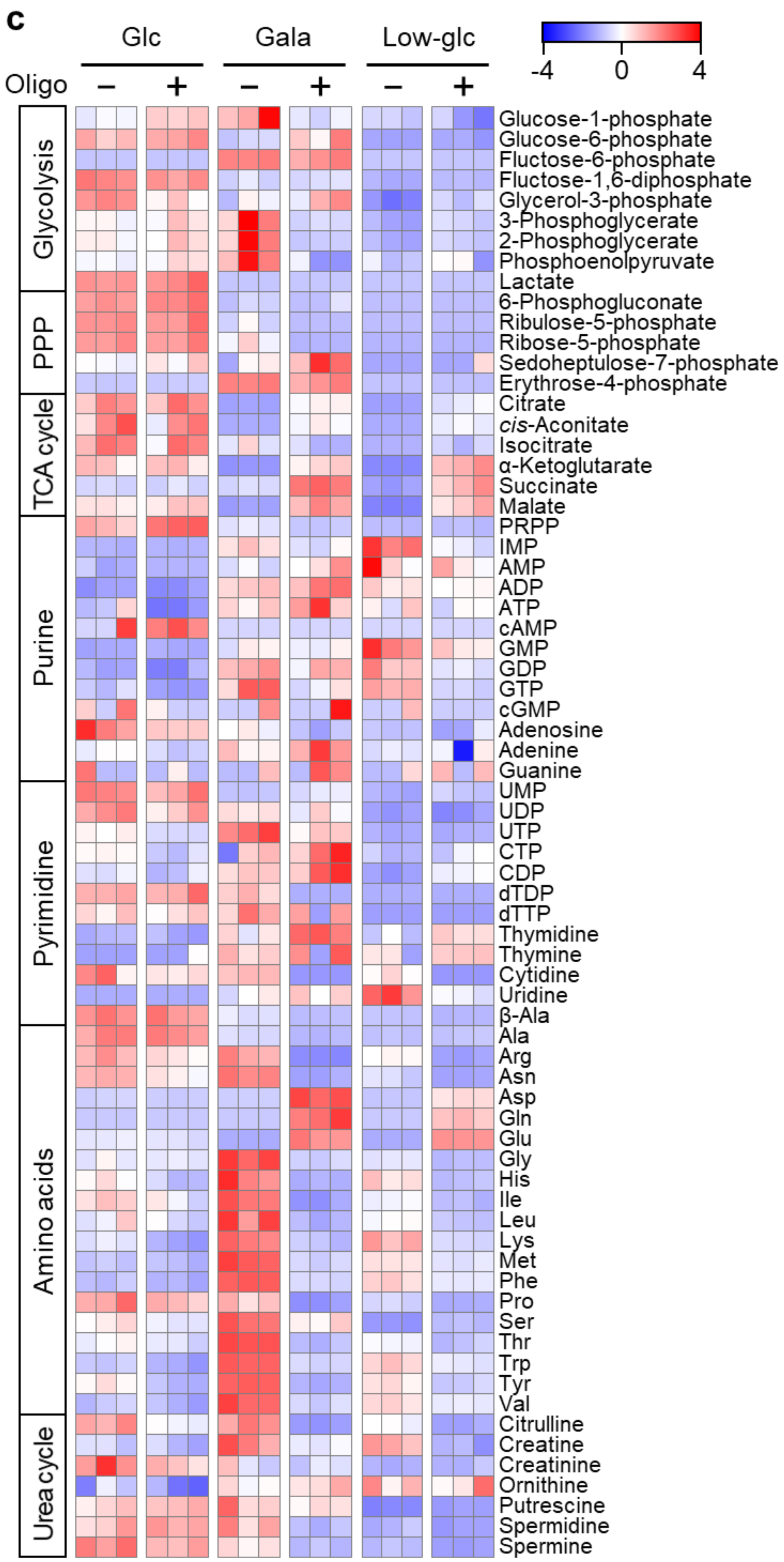
c
Glc
Gala
Low-glc
-4
0
4
Oligo
–
+
–
+
–
+
Glycolysis
PPP
TCA cycle
Purine
Pyrimidine
Amino acids
Urea cycle
Glucose-1-phosphate
Glucose-6-phosphate
Fluctose-6-phosphate
Fluctose-1,6-diphosphate
Glycerol-3-phosphate
3-Phosphoglycerate
2-Phosphoglycerate
Phosphoenolpyruvate
Lactate
6-Phosphogluconate
Ribulose-5-phosphate
Ribose-5-phosphate
Sedoheptulose-7-phosphate
Erythrose-4-phosphate
Citrate
cis-Aconitate
Isocitrate
α-Ketoglutarate
Succinate
Malate
PRPP
IMP
AMP
ADP
ATP
cAMP
GMP
GDP
GTP
cGMP
Adenosine
Adenine
Guanine
UMP
UDP
UTP
CTP
CDP
dTDP
dTTP
Thymidine
Thymine
Cytidine
Uridine
β-Ala
Ala
Arg
Asn
Asp
Gln
Glu
Gly
His
Ile
Leu
Lys
Met
Phe
Pro
Ser
Thr
Trp
Tyr
Val
Citrulline
Creatine
Creatinine
Ornithine
Putrescine
Spermidine
Spermine

Supplementary Fig. S4. Intracellular energy metabolism is reprogrammed to mitochondrial OXPHOS upon glycolytic suppression in PANC-1 cells. (a) Survival rate of PANC-1 cells cultured in glucose (Glc), galactose (Gala), or low-glucose (Low-glc) medium with or without oligomycin (Oligo; 0.8 ng/mL) for 72 hr was evaluated by PI uptake using flow cytometry. Data represent means ± SD of three independent cell cultures. N.S., not significant. **(b)** Oxygen concentration in the culture medium of PANC-1 cells cultured in **(a)** was measured over time. Data represent means ± SD of three independent cell cultures. **(c)** Whole metabolomic patterns of the PANC-1 cells shown in Fig. 3 were visualized using z-score plots and heat maps.

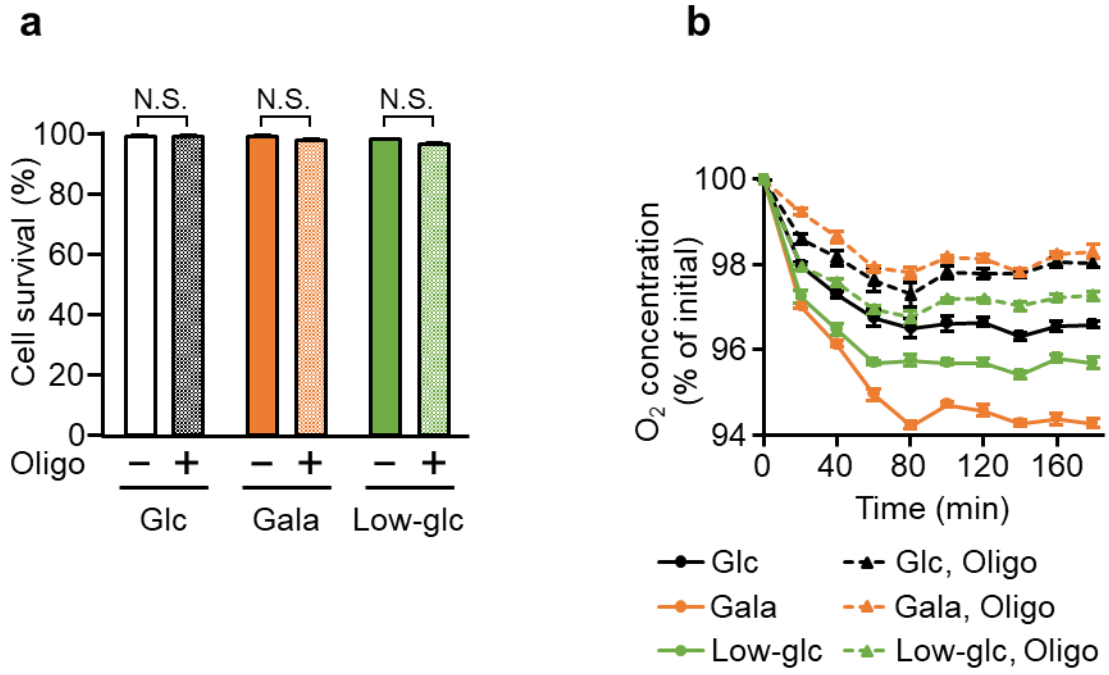
a
N.S.
N.S.
N.S.
Cell survival (%)
100
80
60
40
20
0
Oligo
−
+
−
+
−
+
Glc
Gala
Low-glc
b
O_2 concentration (% of initial)
100
98
96
94
0
40
80
120
160
Time (min)
Glc
Glc, Oligo
Gala
Gala, Oligo
Low-glc
Low-glc, Oligo

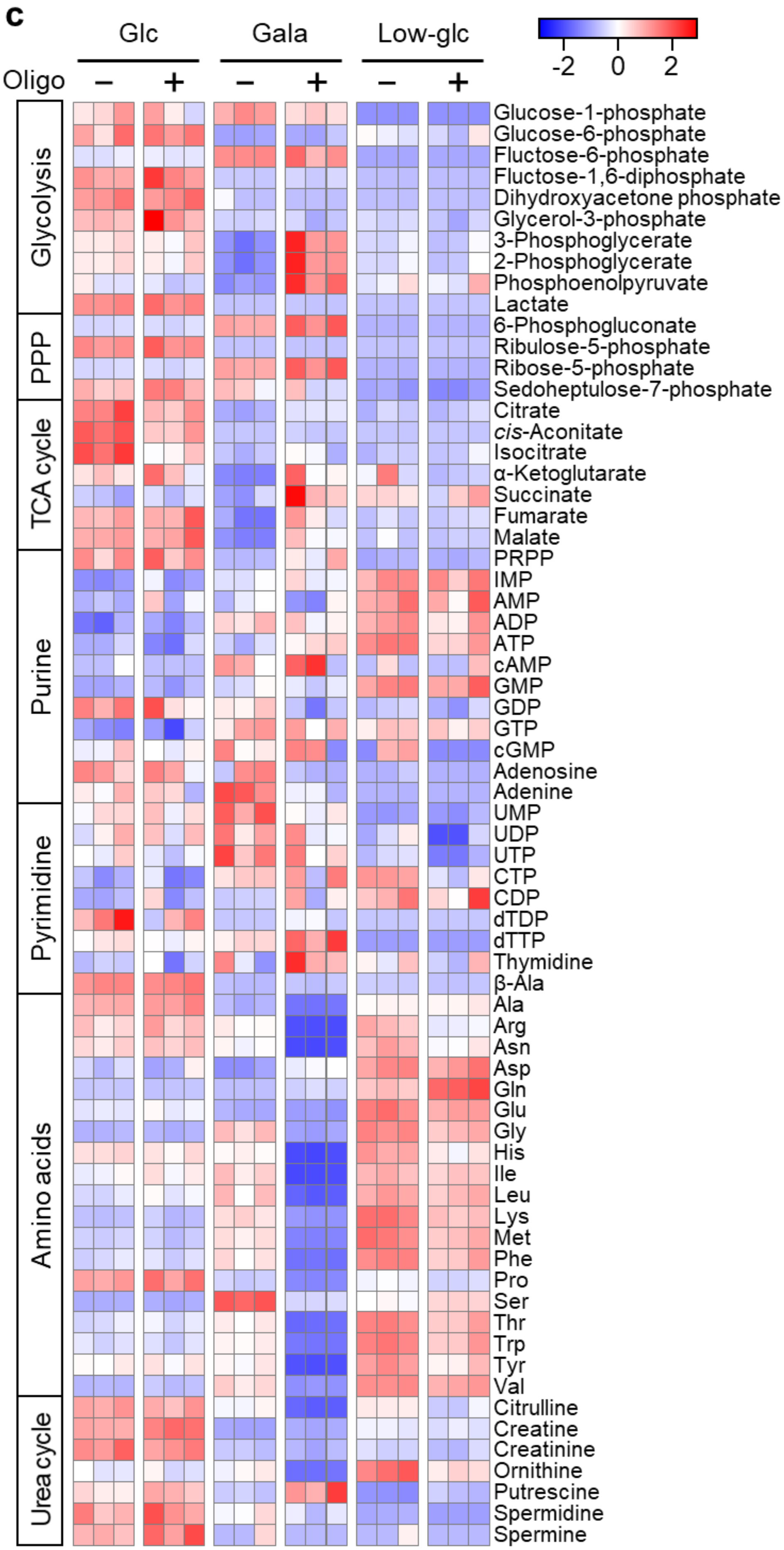

c
Glc
Gala
Low-glc
-2
0
2
Oligo
−
+
−
+
−
+
Glycolysis
PPP
TCA cycle
Purine
Pyrimidine
Amino acids
Urea cycle
Glucose-1-phosphate
Glucose-6-phosphate
Fluctose-6-phosphate
Fluctose-1,6-diphosphate
Dihydroxyacetone phosphate
Glycerol-3-phosphate
3-Phosphoglycerate
2-Phosphoglycerate
Phosphoenolpyruvate
Lactate
6-Phosphogluconate
Ribulose-5-phosphate
Ribose-5-phosphate
Sedoheptulose-7-phosphate
Citrate
cis-Aconitate
Isocitrate
α-Ketoglutarate
Succinate
Fumarate
Malate
PRPP
IMP
AMP
ADP
ATP
cAMP
GMP
GDP
GTP
cGMP
Adenosine
Adenine
UMP
UDP
UTP
CTP
CDP
dTDP
dTTP
Thymidine
β-Ala
Ala
Arg
Asn
Asp
Gln
Glu
Gly
His
Ile
Leu
Lys
Met
Phe
Pro
Ser
Thr
Trp
Tyr
Val
Citrulline
Creatine
Creatinine
Ornithine
Putrescine
Spermidine
Spermine

Supplementary Fig. S5. Metabolic reprogramming by glycolytic suppression also occurs in A549 cells. (a) Survival rate of A549 cells cultured in glucose (Glc), galactose (Gala), or low-glucose (Low-glc) medium with or without oligomycin (Oligo; 1.0 ng/mL) for 72 hr was evaluated by PI uptake using flow cytometry. Data represent means ± SD of three independent cell cultures. N.S., not significant. **(b)** Oxygen concentration in the culture medium of the A549 cells cultured in **(a)** was measured over time. Data represent means ± SD of three independent cell cultures. **(c)** Whole metabolomic patterns of the A549 cells shown in Fig. 4B were visualized using z-score plots and heat maps.

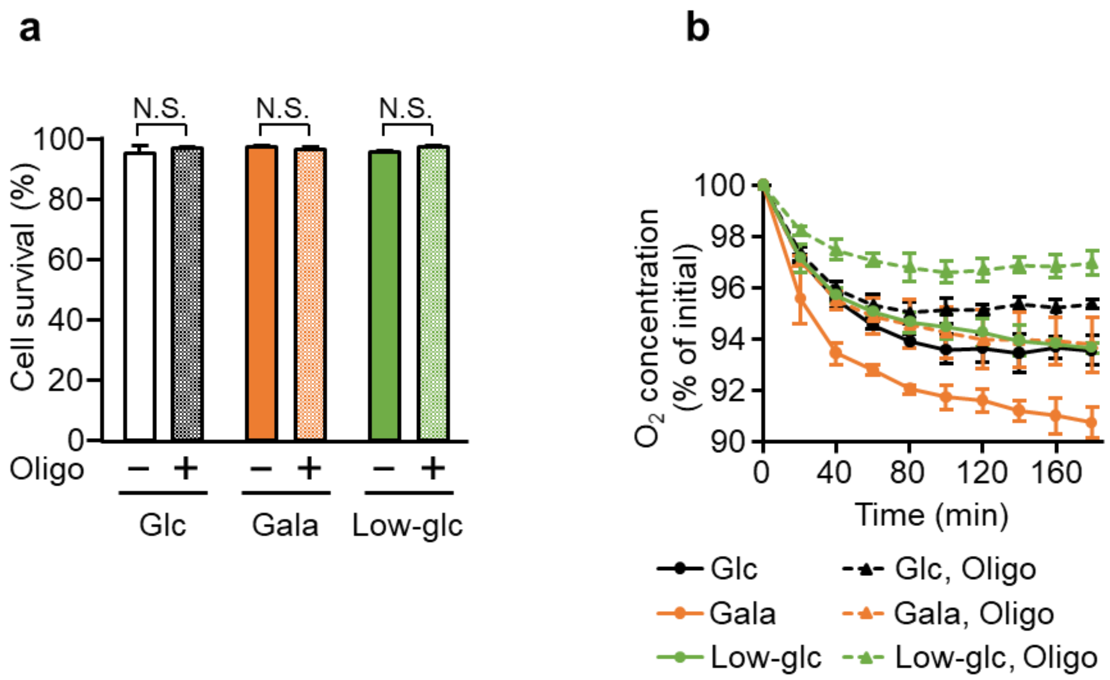
a
N.S.
N.S.
N.S.
Cell survival (%)
100
80
60
40
20
0
Oligo
−
+
−
+
−
+
Glc
Gala
Low-glc
b
O2 concentration (% of initial)
100
98
96
94
92
90
0
40
80
120
160
Time (min)
Glc
Gala
Low-glc
Glc, Oligo
Gala, Oligo
Low-glc, Oligo

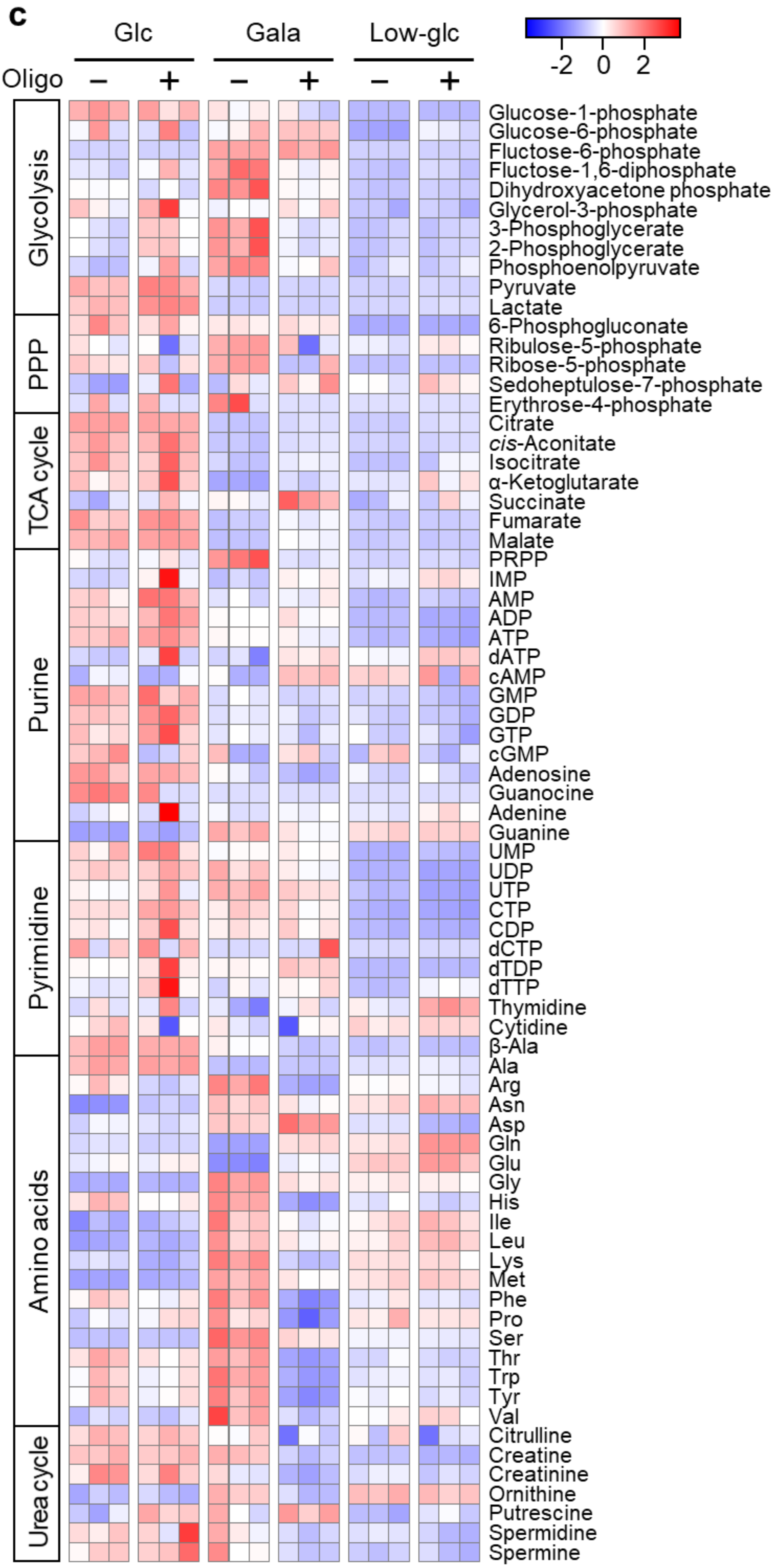
c
Glc
Gala
Low-glc
Oligo
–
+
–
+
–
+
-2
0
2
Glycolysis
PPP
TCA cycle
Purine
Pyrimidine
Amino acids
Urea cycle
Glucose-1-phosphate
Glucose-6-phosphate
Fluctose-6-phosphate
Fluctose-1,6-diphosphate
Dihydroxyacetone phosphate
Glycerol-3-phosphate
3-Phosphoglycerate
2-Phosphoglycerate
Phosphoenolpyruvate
Pyruvate
Lactate
6-Phosphogluconate
Ribulose-5-phosphate
Ribose-5-phosphate
Sedoheptulose-7-phosphate
Erythrose-4-phosphate
Citrate
cis-Aconitate
Isocitrate
α-Ketoglutarate
Succinate
Fumarate
Malate
PRPP
IMP
AMP
ADP
ATP
dATP
cAMP
GMP
GDP
GTP
cGMP
Adenosine
Guanocine
Adenine
Guanine
UMP
UDP
UTP
CTP
CDP
dCTP
dTDP
dTTP
Thymidine
Cytidine
β-Ala
Ala
Arg
Asn
Asp
Gln
Glu
Gly
His
Ile
Leu
Lys
Met
Phe
Pro
Ser
Thr
Trp
Tyr
Val
Citrulline
Creatine
Creatinine
Ornithine
Putrescine
Spermidine
Spermine

Supplementary Fig. S6. Metabolic reprogramming by glycolytic suppression also occurs in HeLa cells. (a) Survival rate of HeLa cells cultured in glucose (Glc), galactose (Gala), or low-glucose (Low-glc) medium with or without oligomycin (Oligo; 0.8 ng/mL) for 72 hr was evaluated by PI uptake using flow cytometry. Data represent means ± SD of three independent cell cultures. N.S., not significant. **(b)** Oxygen concentration in the culture medium of HeLa cells cultured in **(a)** was measured over time. Data represent means ± SD of three independent cell cultures. **(c)** Whole metabolomic patterns of the HeLa cells shown in Fig. 4D were visualized using z-score plots and heat maps.

a

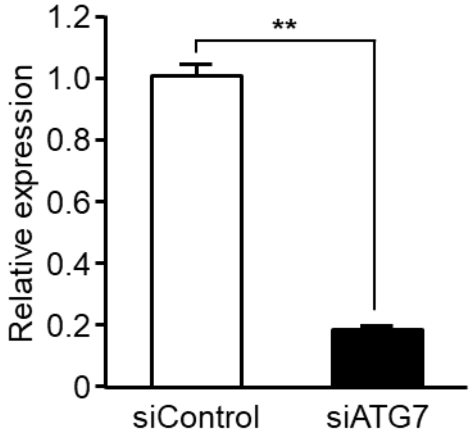
**
1.2
1.0
0.8
0.6
0.4
0.2
0
Relative expression
siControl
siATG7

b

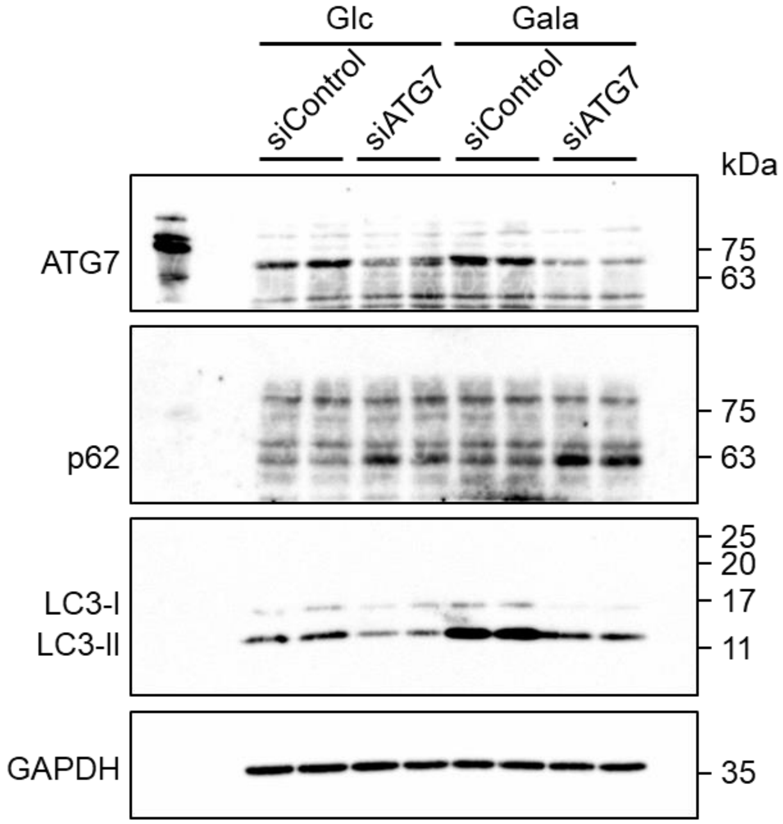
Glc
Gala
siControl
siATG7
siControl
siATG7
kDa
ATG7
75
63
p62
75
63
LC3-I
LC3-II
25
20
17
11
GAPDH
35

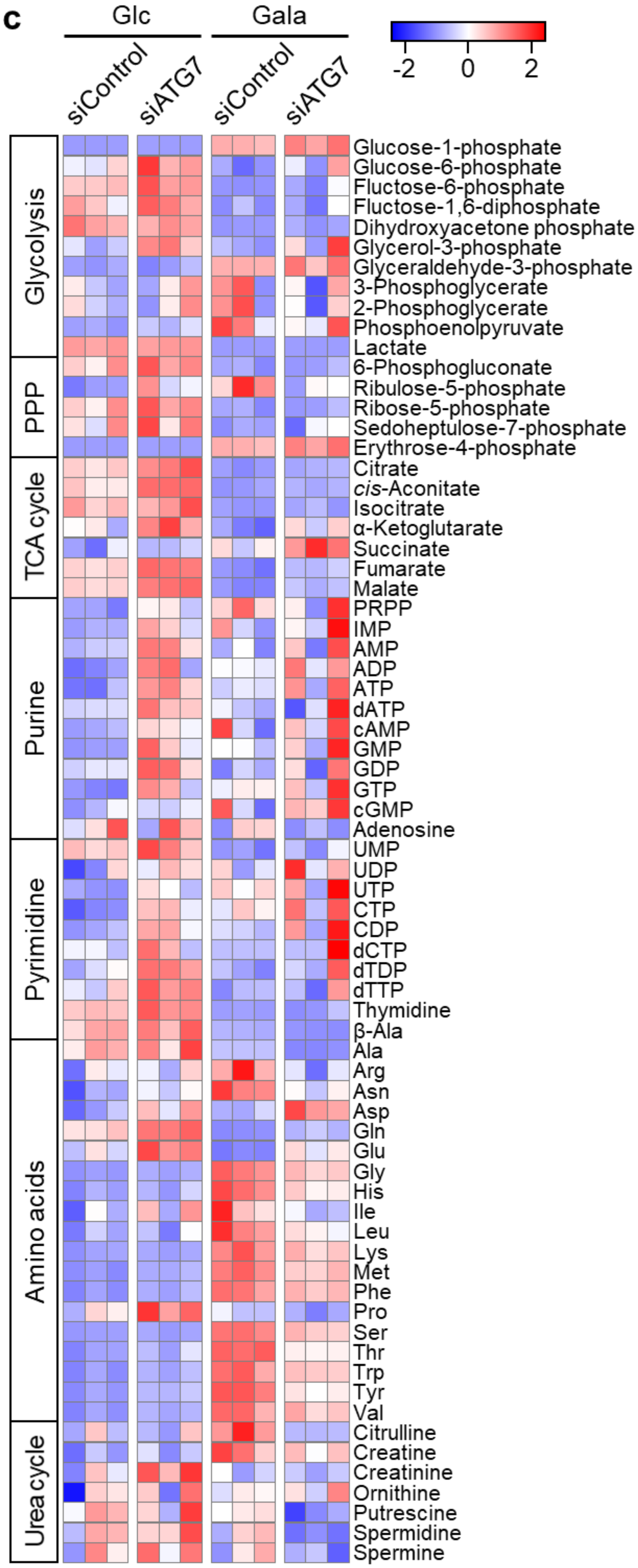
c
Glc
Gala
siControl
siATG7
siControl
siATG7
-2
0
2
Glycolysis
PPP
TCA cycle
Purine
Pyrimidine
Amino acids
Urea cycle
Glucose-1-phosphate
Glucose-6-phosphate
Fluctose-6-phosphate
Fluctose-1,6-diphosphate
Dihydroxyacetone phosphate
Glycerol-3-phosphate
Glyceraldehyde-3-phosphate
3-Phosphoglycerate
2-Phosphoglycerate
Phosphoenolpyruvate
Lactate
6-Phosphogluconate
Ribulose-5-phosphate
Ribose-5-phosphate
Sedoheptulose-7-phosphate
Erythrose-4-phosphate
Citrate
cis-Aconitate
Isocitrate
α-Ketoglutarate
Succinate
Fumarate
Malate
PRPP
IMP
AMP
ADP
ATP
dATP
cAMP
GMP
GDP
GTP
cGMP
Adenosine
UMP
UDP
UTP
CTP
CDP
dCTP
dTDP
dTTP
Thymidine
β-Ala
Ala
Arg
Asn
Asp
Gln
Glu
Gly
His
Ile
Leu
Lys
Met
Phe
Pro
Ser
Thr
Trp
Tyr
Val
Citrulline
Creatine
Creatinine
Ornithine
Putrescine
Spermidine
Spermine

Supplementary Fig. S7. Autophagy mediates metabolic reprogramming toward OXPHOS in glycolysis-suppressed PANC-1 cells. (a) PANC-1 cells transfected with control siRNA (siControl) or siATG7 were subjected to quantitative RT-PCR to measure *ATG7* mRNA levels. Data represent means ± SD of three independent cell cultures. ** $P < 0.01$. **(b)** PANC-1 cells transfected with siControl or siATG7 were cultured in glucose (Glc) or galactose (Gala) medium for 24 hr. The cell lysates were subjected to western blotting with anti-ATG7, anti-p62, anti-LC3B, and anti-GAPDH antibodies. **(c)** Whole metabolomic patterns of the PANC-1 cells shown in Fig. 6C were visualized using z-score plots and heat maps.

Supplementary Materials and Methods

Reagents

2-DG was dissolved in DMSO. The final DMSO concentration in the cell culture did not exceed 0.5% (v/v).

Evaluation of mitochondrial membrane potential and content

To evaluate mitochondrial membrane potential and content, PANC-1 cells were incubated for 30 minutes with 125 nM MitoTracker Orange and 125 nM MitoTracker Green (both from Life Technologies) dissolved in FBS-free RPMI-1640. After loading, cells were washed, and images were acquired on a Carl Zeiss LSM700 laser scanning confocal microscope.

RNA isolation and quantitative real-time PCR

Total RNA was isolated from PANC-1 cells using the Sepasol-RNA I reagent (Nacalai Tesque) and reverse-transcribed using ReverTra Ace qPCR RT Master mix (TOYOBO). The resulting cDNA was mixed with THUNDERBIRD quantitative real-time PCR mix. The mixture was subjected to quantitative real-time PCR as described in Materials & Methods. Primers were as follows: *ATG7* forward, 5′-ACC CAG AAG AAG CTG AAC GA-3′; reverse, 5′-CTG CTT GTT CCA AAA GGA GC-3′; *β-actin* forward, 5′-TTC AAC ACC CCA GCC ATG TAC G-3′; reverse, 5′-GTG GTG GTG AAG CTG TAG CC-3′.

Western blotting

PANC-1 cells were lysed on ice in lysis buffer [PBS (pH 7.4) containing 1% Triton X-100 and protease inhibitor cocktail (Roche)]. Identical amounts of protein from each sample were separated by SDS-PAGE and transferred to a PVDF membrane (Merck Millipore, Berlin, Germany). Membranes were blocked and probed with primary antibodies specific for MDH2, GDH1/2, ATG7, p62, LC3B, and GAPDH (all from Cell Signaling Technology). Immunolabeled proteins were detected using HRP-labeled secondary antibodies (Santa Cruz Biotechnology, Dallas, TX, USA) and ECL Prime detection reagents (GE Healthcare, Buckinghamshire, UK). Signals were visualized using the ImageQuant LAS 4000 system (GE Healthcare).

Quantitative proteomics analysis

In vitro proteome-assisted multiple reaction monitoring for protein absolute quantification (iMPAQT) analysis was performed at Kyushu Pro Search LLP (Fukuoka, Japan) [1].

Supplementary Reference

1 Matsumoto, M. *et al.* A large-scale targeted proteomics assay resource based on an in vitro human proteome. *Nat Methods* **14**, 251-258, doi:10.1038/nmeth.4116 (2017).
